# The impact of supplementing vitamin D through different methods on the prognosis of COVID-19 patients: a systematic review and meta-analysis

**DOI:** 10.3389/fnut.2024.1441847

**Published:** 2024-09-25

**Authors:** Xiangqun Zhang, Junyuan Wu, Hongmeng Dong, Na Shang, Yixuan Li, Ying Zhang, Shubin Guo, Xue Mei

**Affiliations:** ^1^Emergency Medicine Clinical Research Center, Beijing Chao-Yang Hospital, Capital Medical University, Beijing, China; ^2^Beijing Key Laboratory of Cardiopulmonary Cerebral Resuscitation, Clinical Center for Medicine in Acute Infection, Capital Medical University, Beijing, China

**Keywords:** COVID-19, vitamin D, prognosis, administration methods, dosage, baseline vitamin D

## Abstract

**Objective:**

To analyze the impact of different methods of Vitamin D administration on the prognosis of COVID-19 patients.

**Methods:**

A comprehensive literature search was conducted across four databases: PubMed, Embase, Web of Science, and Cochrane, up to January 5, 2024. Eligible studies included randomized controlled trials and cohort studies that compared Vitamin D supplementation with control groups in COVID-19 patients. Outcomes of interest were mortality rate, ICU (Intensive Care Unit) admission rate, length of hospital stay, and endotracheal intubation rate. Subgroup analyses were performed based on the dosing regimen (single-dose vs. continuous-dose), total Vitamin D intake within 14 days (≥100,000 IU vs. <100,000 IU), and baseline serum Vitamin D levels (deficient group: 25OHD < 30 ng/mL vs. non-restricted group). A random-effects model was employed for meta-analysis to account for heterogeneity among studies.

**Results:**

A total of 21 studies involving 4,553 participants were included. In terms of mortality, Vitamin D supplementation significantly reduced the mortality rate (RR = 0.72, 95% CI: 0.54–0.94, *I*^2^ = 54%, *p* = 0.02), with continuous dosing being more effective (RR = 0.53, 95% CI: 0.34–0.83, *I*^2^ = 55%, *p* = 0.006) compared to single-dose (RR = 0.88, 95% CI: 0.69–1.12, *I*^2^ = 21%, *p* = 0.3), and lower total doses (<100,000 IU) showing greater benefit (RR = 0.30, 95% CI: 0.21–0.44, *I*^2^ = 0%, *p* < 0.0001). Mortality was significantly reduced in the Vitamin D-deficient group (25OHD < 30 ng/mL) (RR = 0.73, 95% CI: 0.59–0.89, *I*^2^ = 0%, *p* = 0.002) but not in the non-restricted group. Regarding ICU admission, supplementation reduced ICU admission rates (RR = 0.58, 95% CI: 0.38–0.88, *I*^2^ = 74%, *p* = 0.01), with continuous dosing (RR = 0.44, 95% CI: 0.22–0.90, *I*^2^ = 74%, *p* = 0.02) being more effective than single-dose (RR = 0.79, 95% CI: 0.61–1.03, *I*^2^ = 22%, *p* = 0.08), and lower doses (<100,000 IU) providing more significant reduction (RR = 0.31, 95% CI: 0.21–0.47, *I*^2^ = 0%, *p* = 0.001). ICU admission rates were significantly reduced in the Vitamin D-deficient group (RR = 0.63, 95% CI: 0.42–0.93, *I*^2^ = 0%, *p* = 0.02) but not in the non-restricted group (RR = 0.59, 95% CI: 0.32–1.11, *I*^2^ = 86%, *p* = 0.1). For length of hospital stay, no significant differences were observed between Vitamin D and control groups (MD = −1, 95% CI: −2.16 to 0.16, *p* = 0.13), and subgroup analyses by dosing regimen, total dose, and baseline Vitamin D levels also showed no significant differences. Similarly, for endotracheal intubation, there was no significant difference in intubation rates between groups (RR = 0.78, 95% CI: 0.56–1.08, *p* = 0.13), and subgroup analyses confirmed no significant effect of different dosing strategies or baseline Vitamin D status on intubation rates.

**Conclusion:**

Vitamin D supplementation improves clinical outcomes in COVID-19 patients by reducing mortality and ICU admission rates, particularly when administered continuously with a total dose of less than 100,000 IU over 14 days, and among those with baseline Vitamin D deficiency (25OHD < 30 ng/mL). However, there were no significant effects on the length of hospital stay or endotracheal intubation rates, regardless of the dosing regimen or baseline Vitamin D levels. These findings emphasize the importance of considering both the total dose over 14 days and baseline Vitamin D status to optimize therapeutic benefits.

## 1 Introduction

Coronavirus Disease 2019 (COVID-19) is a systemic respiratory disease caused by the novel coronavirus (Severe Acute Respiratory Syndrome Coronavirus 2:SARS-CoV-2). Since December 2019, COVID-19 has spread globally, affecting millions of people and resulting in hundreds of thousands of deaths. Significant progress has been made in the prevention and treatment of COVID-19 using effective vaccines and antiviral drugs. However, intermittent outbreaks of the novel coronavirus continue worldwide, posing a threat to human health and life. Thus, there is a need to explore effective preventive and therapeutic drugs to aid epidemic control. Previous studies have stated that Vitamin D enhances innate and cellular immunity (1–3) and reduces the survival and replication of respiratory viruses. Besides, numerous studies have established an association between low Vitamin D levels and an increased risk of acute respiratory virus infections (4, 5), which can be reduced through Vitamin D supplementation (6, 7). Moreover, Vitamin D supplementation has also been associated with a reduction in all-cause mortality (8). Since the COVID-19 pandemic began, some studies and meta-analyses have confirmed an association between low blood Vitamin D levels and adverse outcomes in patients with novel coronavirus infection (9–13). Nevertheless, research results regarding using Vitamin D supplementation to improve outcomes in patients with COVID-19 infection are not clear. Previous systematic reviews and meta-analyses of RCTs (randomized controlled trials) have yielded inconsistent results. Some RCT studies have asserted that supplementation with Vitamin D shortens the recovery time for mild to moderate COVID-19 symptoms such as cough and loss of taste (14). Furthermore, there have been reports of reduced severity (length of hospital stay, need for oxygen or respiratory support, etc.) and mortality rates (15–19). However, these findings are inconsistent, since other studies have concluded that supplementation with Vitamin D does not improve mortality rates or any other severity indicators in COVID-19 patients, including the need for endotracheal intubation and length of hospital stay (20–24).

By pooling results from diverse studies, we aim to provide a clearer understanding of the overall impact of Vitamin D supplementation on COVID-19 outcomes. This approach not only enhances the statistical power and generalizability of our findings but also helps to identify patterns and factors that may explain the variability in individual study results. Specifically, this review examines whether immediate Vitamin D supplementation upon hospital admission can improve the prognosis of COVID-19 patients. By comparing the outcomes across various target populations, dosages, and methods of Vitamin D supplementation, we aim to identify the most effective strategies for administering Vitamin D to these patients.

## 2 Research methods

This systematic review and meta-analysis is reported according to the Preferred Reporting Items for Systematic Reviews and Meta-Analyses (PRISMA) guidelines (25) and is registered in PROSPERO website: https://www.crd.york.ac.uk/prospero/ID:CRD42024545945, PROSPERO Registration No: CRD42024545945.

### 2.1 Search strategy

Searches were conducted in the PubMed, Embase, Web of Science, and Cochrane databases, with search dates ranging from inception to January 5, 2024. The search strategy followed the PICOS principles, primarily focusing on the study population, intervention methods, and research methodology. The search terms and keywords used were: “COVID-19,” “2019-nCoV Infection,” “infection 2019-nCoV,” “SARS-CoV-2 Infection,” “SARS CoV 2 Infection,” “2019 Novel Coronavirus Disease,” “2019 Novel Coronavirus Infection,” “COVID-19 Virus Infections,” “Infection COVID-19 Virus,” “Virus Infection COVID-19,” “Coronavirus Disease 2019,” “Disease 2019, Coronavirus,” “Coronavirus Disease 19,” “Severe Acute Respiratory Syndrome Coronavirus 2 Infection,” “COVID-19 Virus Disease,” “Disease COVID-19 Virus,” “Virus Disease, COVID-19,” “SARS Coronavirus 2 Infection,” “2019 nCoV Disease,” “Disease, 2019-nCoV,” “COVID-19 Pandemic,” “Pandemic, COVID-19,” “Vitamin D,” “Calciol,” “Vitamin D 3,” “Cholecalciferol,” “25 HydroxyVitamin D3,” “Calcidiol,” “25 Hydroxycholecalciferol,” “Calcifediol,” “Dedrogyl,” “Hydropherol,” and “Calderol.” The specific search strings are provided in Supplementary Table S1. To supplement the research, manual searches were conducted by retrieving bibliographies of relevant reviews and identified articles. If necessary, contact was made with the study authors to obtain additional information.

### 2.2 Inclusion and exclusion criteria

The inclusion criteria for this meta-analysis were based on the following Population, Intervention, Comparison, Outcomes, and Study design (PICOS) criteria: (1) Participants: admitted patients aged ≥18 years with confirmed COVID-19 diagnosis; (2) Intervention: supplementation with Vitamin D; (3) Comparison: no Vitamin D supplementation or lower-dose supplementation; (4) Outcome: mortality, ICU admission, length of hospital stay, or need for endotracheal intubation; (5) Study design: randomized controlled trials or observational studies. Certain articles including reviews, simulation studies, animal studies, letters, conference papers, and case studies were all excluded from this study.

### 2.3 Data extraction

Data extraction was performed independently by two researchers (Yixuan LI and Ying Zhang). Any discrepancies were resolved by referring to the third author, Zhang Xiangqun, if necessary. The following information was extracted from all eligible studies: first author’s name, publication year, country of study, study design, patient characteristics, intervention methods and dosage, and clinical outcomes (mortality rate, ICU admission rate, length of hospital stay, and rate of endotracheal intubation). Data from included studies were entered into a dedicated spreadsheet using Microsoft Excel (Microsoft Corporation, Redmond, WA, United States). In cases of missing data regarding the primary outcomes, we contacted the corresponding authors of the original studies.

### 2.4 Statistical analysis

All analyses were conducted using Review Manager software version 5.4 (Nordic Cochrane Center, Cochrane Collaboration). A significance level of *p* < 0.05 (two-tailed) was used for all statistical tests. For binary data, risk ratios (OR) and their 95% confidence intervals (CI) were calculated. For continuous data, mean differences (MD) and their 95% CIs were calculated.

In assessing heterogeneity, we selected the model based on its extent. A fixed-effects model was employed when the variability across studies was minimal, suggesting similar effect sizes. Conversely, a random-effects model was used when substantial variability was present, reflecting diverse effect sizes. This approach not only considers the degree of heterogeneity but also accounts for study characteristics such as design, sample demographics, intervention specifics, outcome measurement methods, and contextual factors. By integrating these considerations, we aimed to accurately reflect both within-study and between-study variations, ensuring a robust and reliable overall effect estimate.

If the effect size was represented by standardized mean differences (MD) along with 95% CIs, the mean (26) and standard deviation (27) of the quartile data were calculated using the provided formulas. Publication bias for each study was assessed by constructing funnel plots of the effect size against standard error. Sensitivity analysis was conducted by systematically excluding individual studies to assess the robustness of the results. Subgroup treatment effects were compared using Cochran’s Q test and Higgins’s I^2^ statistic, with *p* < 0.05 indicating a statistically significant difference.

### 2.5 Assessment of study quality

The quality of the included literature was assessed by author Hongmeng Dong and Na Shang according to the quality assessment criteria of the Cochrane Collaboration (28). When including randomized controlled trials, the Cochrane Handbook recommends using a revised version of the Cochrane tool, known as the Risk of Bias tool (RoB 2) (29). The RoB 2 tool provides a framework for assessing the risk of bias for individual outcomes in any type of randomized trial. Evaluation criteria include random sequence generation, allocation concealment, participant and personnel blinding, outcome assessment blinding, incomplete outcome data, selective reporting, and other biases. In this study, the reviewers assessed different studies based on the Cochrane Handbook guidelines. The risk of bias for each domain can be categorized into three levels: “low risk,” “some concerns,” and “high risk.” If all domains are assessed as low risk, the overall risk of bias is low. If one or more domains are judged as “some concerns” but none are assessed as high risk, the overall risk of bias is “some concerns.” If at least one domain is evaluated as high risk, the overall risk of bias is considered “high risk” (30).

## 3 Results

### 3.1 Study selection

**Figure 1:**
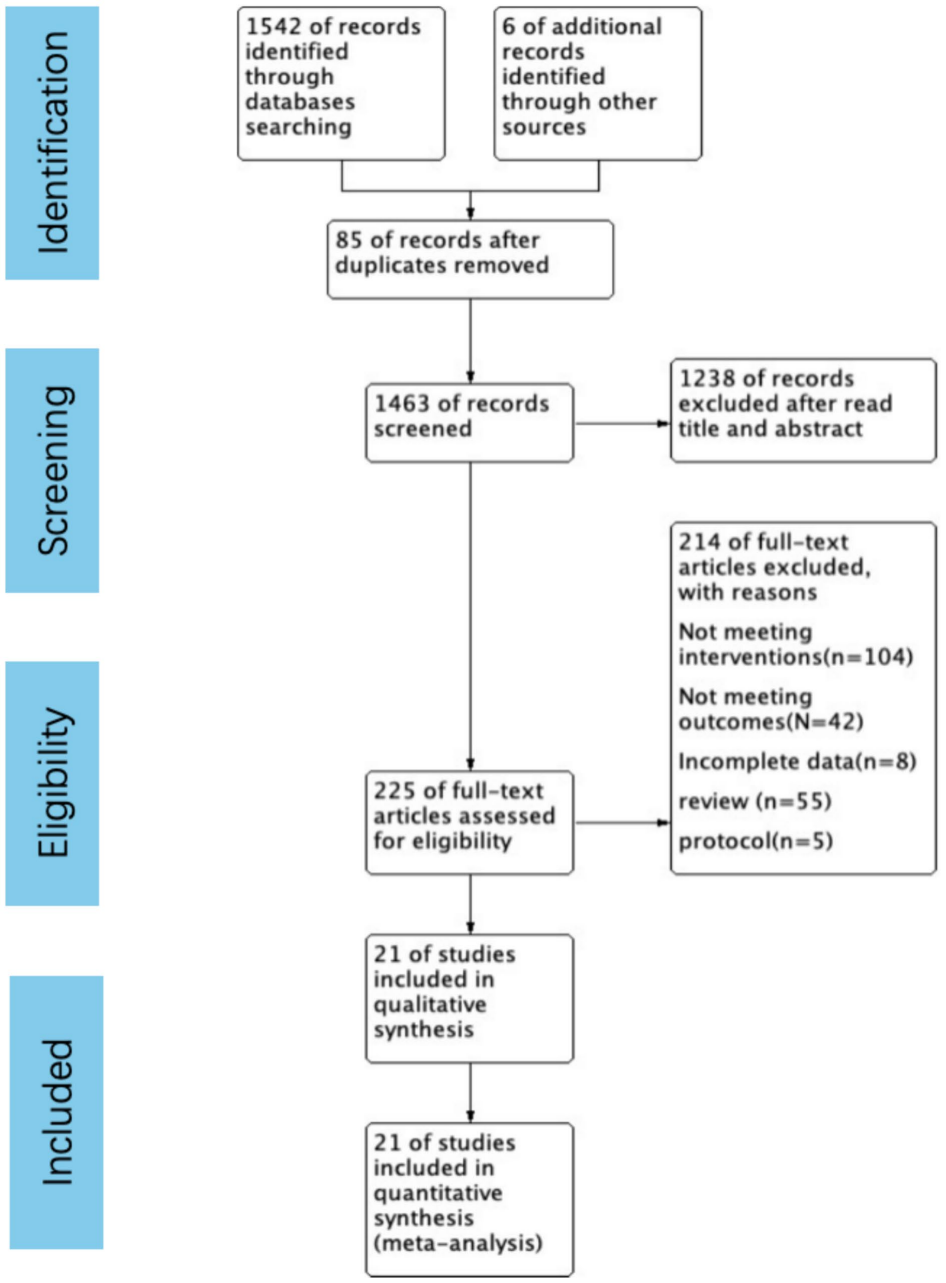
PRISMA flow chart at four levels.

The systematic review, selection, and exclusion criteria are summarized in Figure 1. A total of 1,548 articles were retrieved, and after removing duplicate publications, 1,463 articles were included for analysis. After carefully reviewing the titles and abstracts, 225 articles remained. The full texts were all read thoroughly, and then a final selection of 21 articles (14, 16–21, 23, 24, 31–42) was made. These studies included a total of 4,553 patients, with 2,164 patients in the Vitamin D supplementation groups and 2,389 patients in the control groups.

### 3.2 Study characteristics

There were a total of 21 studies from Spain (*n* = 5), Turkey (*n* = 2), Brazil (*n* = 2), France (*n* = 1), Argentina (*n* = 1), Croatia (*n* = 1), Egypt (*n* = 1), Saudi Arabia (*n* = 1), Belgium (*n* = 1), Italy (*n* = 1), the United States (*n* = 1), Ireland (*n* = 1), Russia (*n* = 1), Thailand (*n* = 1), and India (*n* = 1). Among them, 16 were randomized controlled trials and five were cohort studies.

Of the 4,553 participants, 2,164 were in intervention groups and 2,389 were in control groups. The ages of the participants ranged from 31.1 to 93 years. Nineteen studies reported on participant mortality (14, 16–21, 23, 24, 31, 32, 34–36, 38–42), involving 4,115 participants. Thirteen studies reported on ICU admission rates (14, 17, 19, 21, 23, 31, 35, 36, 38–41) and included 2,811 participants. There were 15 studies involving 2,652 participants that assessed the length of hospital stay (14, 17–21, 23, 24, 32–35, 37, 39, 41). Ten studies reported on intubation rates (16, 20, 21, 31–33, 38, 39, 41, 42), with 1,995 participants. Among the 21 clinical trials, the intervention groups received varying daily doses of Vitamin D, ranging from 20 IU to 600,000 IU. In contrast, the control groups in two clinical trials received small doses of Vitamin D orally, relative to the intervention group. The remaining 19 trials used either a placebo or no intervention. In eight clinical trials, the intervention group members were administered Vitamin D as a single dose upon admission, while the remaining 13 trials involved continuous Vitamin D supplementation. The intervention group members of 13 clinical trials received a total Vitamin D dose of greater than or equal to 100,000 IU in the 14 days after hospitalization, while the patients in the remaining eight trials received a total dose of less than 100,000 IU. Table 1 offers further details of the study characteristics.

**Table 1:** Study characteristics.

Author/year of publication	Study design	Nationality	Participants	Intervention group	Control group	Age intervention group	Age control group	Intervention group female *n* (%)	Control group female *n* (%)	Intervention group: Control group (vitamin D supplement method)	Outcomes	Author/year of publication	Study design
Ajay Singh/2024	RCT/NCT04952857	India	90	45	45	54.54 ± 21.44	46.71 ± 12.52	18 (40)	17 (38)	600,000 IU:0	Mortality/tracheal intubation	Ajay Singh/2024	RCT/NCT04952857
Alan L Fernandes/2022	RCT/NCT04449718	Brazil	144	71	73	55.3 ± 14.2	55.7 ± 14.5	34 (47.9)	33 (45.2)	200,000 IU:0	LOS	Alan L Fernandes/2022	RCT/NCT04449718
Ce ´dric Annweiler/2022	RCT/NCT04344041	France	244	122	122	87 (81–92)	89 (83–93)	66 (52)	82 (65)	400,000I:50,000 IU	Mortality/ICU/LOS	Ce ´dric Annweiler/2022	RCT/NCT04344041
Igor H. Murai/2021	RCT/NCT04449718	Brazil	237	119	118	55.7 ± 16.6	61.3 ± 14.4	7 (43.8)	10 (62.5)	200,000 IU:0	Mortality/ICU/LOS/tracheal intubation	Igor H. Murai/2021	RCT/NCT04449718
Javier Mariani/2021	RCT/NCT04411446	Argentina	218	115	103	59.8 ± 10.7	58.3 ± 10.6	51 (44.3)	52 (50.5)	500,000 IU:0	Mortality/ICU/LOS/tracheal intubation	Javier Mariani/2021	RCT/NCT04411446
Jorge B Cannata‐Andía/2022	RCT/NCT04552951	Spain	543	274	269	59.0 [49.-70]	57.0 [45–67]	93 (33.9)	97 (36.1)	100,000 IU:0	Mortality/ICU/LOS	Jorge B Cannata‐Andía/2022	RCT/NCT04552951
Josipa Domazet Bugarin/2023	RCT/NCT05384574	Croatia	152	75	77	65 (59–71)	65.5 (39–82)	23 (30.7)	19 (25.5)	10,000*14 (at least):0	Mortality/length of LOS	Josipa Domazet Bugarin/2023	RCT/NCT05384574
Juan F. Alcala-Diaz/2021	Cohort study/NA	Spain	537	79	458	69 ± 15	67 ± 16	398 (86.9)	26 (32.9)	D1:21280IU; D3, D7:10640IU;10640IU/W:0	Mortality/tracheal intubation	Juan F. Alcala-Diaz/2021	Cohort study/NA
Marta Entrenas Castillo/2020	RCT/NCT04366908	Spain	76	50	26	53.14 ± 10.77	52.77 ± 9.35	23 (46%)	8 (31%)	D1:21280IU; D3, D7:10640IU;10640IU/W:0	Mortality/ICU	Marta Entrenas Castillo/2020	RCT/NCT04366908
Mehmet Güven/2021	Cohort study/NA	Türkiye	175	113	62	74 (60–81)	75 (62–83)	44 (39%)	26 (42%)	300,000 IU:0	Mortality/length of LOS	Mehmet Güven/2021	Cohort study/NA
Miguel Cervero /2022	RCT (pilot study)/ID01052020	Spain	85	41	44	64 (44–72)	67 (58–75)	14 (32%)	11 (27%)	10,000 IU/D:2000 U/D	Mortality/ICU/LOS/tracheal intubation	Miguel Cervero/2022	RCT (pilot study)/ID01052020
MikhailV. Bychinin/2022	RCT/NCT05092698	Russia	106	52	54	64.5 (57–71)	63.5 (54–81)	30 (54.5)	23 (42.59)	D1:60,000IU, D2–14:5,000单位/天:0	Mortality/length of LOS/tracheal intubation	MikhailV. Bychinin/2022	RCT/NCT05092698
Mustafa Sait Gönen/2021	Cohort study/NA	Türkiye	314	163	151	55.00 ± 16.45	50.23 ± 12.36	52 (34.4%)	80 (49.4%)	D1:100,000 IU; D2–14 10,000 IU/5,000 IU/2,000 IU:0	Mortality/ICU/LOS	Mustafa Sait Gönen/2021	Cohort study/NA
Neven Sarhan/2022	RCT/NCT04738760	Egypt	116	58	58	66.1 ± 11.2	65.7 ± 12.6	20 (34.5%)	12 (20.7%)	200,000 IU:40 IU/D	Mortality/ICU/LOS/tracheal intubation	Neven Sarhan/2022	RCT/NCT04738760
Pitchaya Dilokpattanamongkol/2024	RCT/TCTR20210906005	Thailand	294	147	147	47.90 ± 16.77	53.71 ± 18.80	85 (57.80)	72 (49.00)	80 IU/D*14:0	LOS/tracheal intubation	Pitchaya Dilokpattanamongkol/2024	RCT/TCTR20210906005
Shaun Sabico/2021	RCT/SCTR20061006	Saudi Arabia	69	36	33	46.3 ± 15.2	53.5 ± 12.3	20 (60.6%)	15 (41.7%)	5,000 IU/D*14:1,000 IU/D*14	Mortality/ICU/LOS	Shaun Sabico/2021	RCT/SCTR20061006
Sophie De Niet/2022	RCT/NCT04636086	Belgium	43	21	22	63.24 ± 14.46	68.73 ± 10.97	8 (38%)	12 (54%)	25,000 IU/D*4, D5–14 25,000/W:0	Mortality/ICU/LOS	Sophie De Niet/2022	RCT/NCT04636086
Vito Fiore/2022	Cohort study/NA	Italy	116	58	58	62.5 ± 14.8	62.9 ± 12.8	25 (22.1)	25 (22.1)	100,000 IU/D*2d:0	Mortality/ICU/tracheal intubation	Vito Fiore/2022	Cohort study/NA
Xavier Nogues/2021	Cohort study/NA	Spain	838	447	391	61.81 ± 15.5	62.41 ± 17.2	183 (40.9%)	160 (40.9%)	D1:21280IU:D3, D7:10640IU; 10640IU/W:0	Mortality/ICU	Xavier Nogues/2021	Cohort study/NA
Yasmine M. Elamir/2022	RCT (pilot study)/NA	The United States	50	25	25	69 ± 18	64 ± 16	13 (52%)	12 (48%)	20 IU/D*14:0	Mortality/ICU/LOS	Yasmine M. Elamir/2022	RCT (pilot study)/NA
Zhila Maghbooli/2021	RCT/NCT04386850	Ireland	106	53	53	50 ± 15	49 ± 13	41% (22) 38% (20)	41% (22)	1,000 IU/D*14:0	Mortality/ICU/LOS/tracheal intubation	Zhila Maghbooli/2021	RCT/NCT04386850

LOS, Length of Hospital Stay; ICU, Intensive Care Unit.

### 3.3 Risk of bias

#### 3.3.1 Methods section

To assess the risk of bias in the included studies, we used the Cochrane Risk of Bias (RoB) assessment tool, which evaluates six domains of bias: selection bias, performance bias, detection bias, attrition bias, reporting bias, and other biases. Disagreements during the assessment process were resolved through discussion, and if consensus could not be reached, arbitration was conducted by a third reviewer. We also performed sensitivity analyses to evaluate the robustness of the study results, including the exclusion of studies with high risk of bias and studies with extreme effect sizes or outliers. Meta-analyses were conducted using RevMan 5.3 software, generating an overall risk of bias graph.

#### 3.3.2 Results section

**Figure 2:**
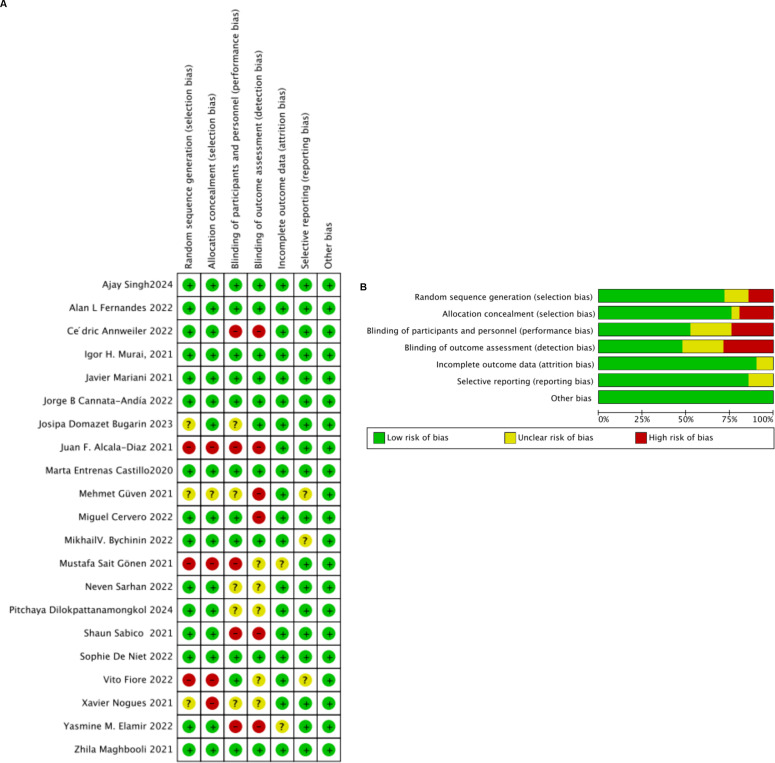
**(A)** Risk of bias summary: review authors’ judgments about each risk of bias item for each included study. **(B)** Risk of bias graph: review authors’ judgments about each risk of bias item presented as percentages across all included studies.

Among the 21 clinical trials included, random sequence generation in 15 randomized controlled trials (RCTs) was deemed low risk of bias, while three cohort studies were classified as high risk, and three were uncertain. Regarding allocation concealment, 16 studies were considered low risk, one did not report allocation methods and was thus deemed uncertain, and four were classified as high risk. Four RCTs did not employ blinding, four did not mention blinding, and none of the cohort or observational studies applied blinding. The risk of bias in outcome blinding assessment was generally low in the RCTs; specifically, 11 studies conducted double-blind trials, 5 did not implement double-blinding, and 5 did not clearly specify their blinding procedures. In cohort and observational studies, outcome blinding was not explicitly mentioned. A detailed summary of these findings is provided in Figures 2A,B.

#### 3.3.3 Sensitivity analysis

We performed sensitivity analyses to evaluate the robustness of our findings by excluding studies with a high risk of bias or outliers. The results indicated that the main outcomes, such as mortality and ICU admission rates, remained consistent even after excluding these high-risk or extreme studies. This suggests that our findings are robust and not influenced by individual studies with a high risk of bias or extreme values.

### 3.4 Meta-analyses

#### 3.4.1 Effect of oral vitamin D on the mortality rate of COVID-19-infected individuals

**Figure 3:**
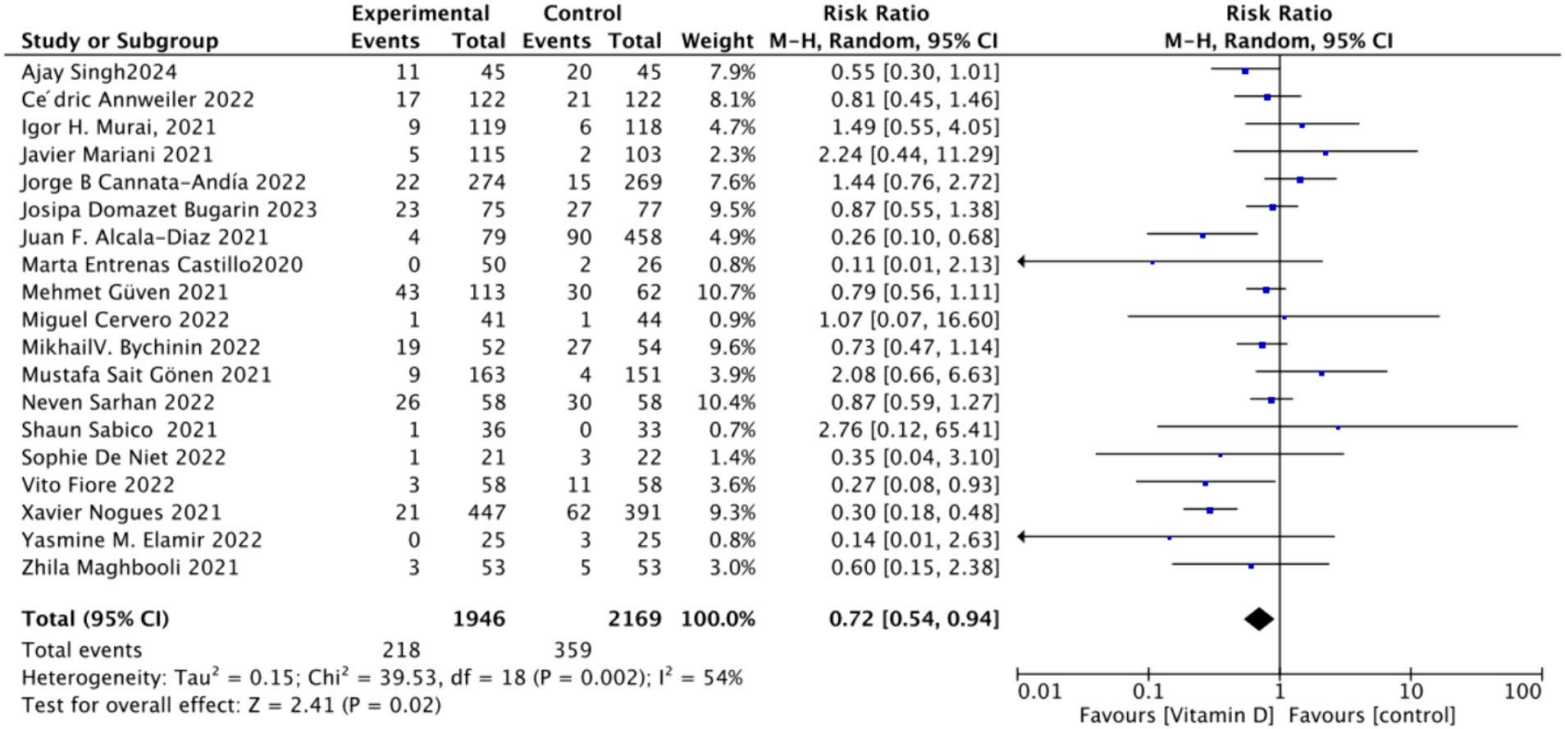
Forest plot of mortality rate.

**Figure 4:**
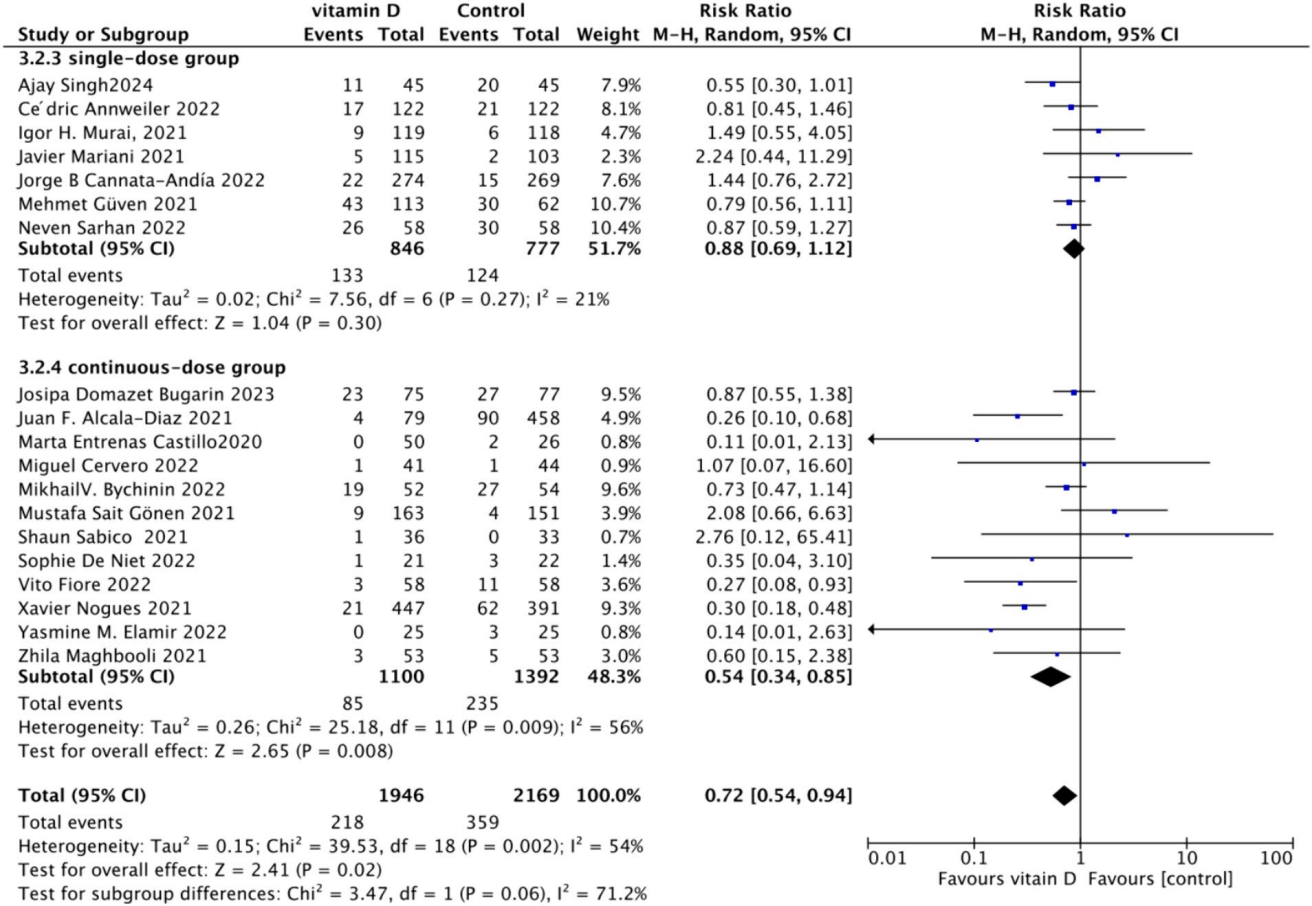
Forest plot of mortality rate by administration method subgroup.

**Figure 5:**
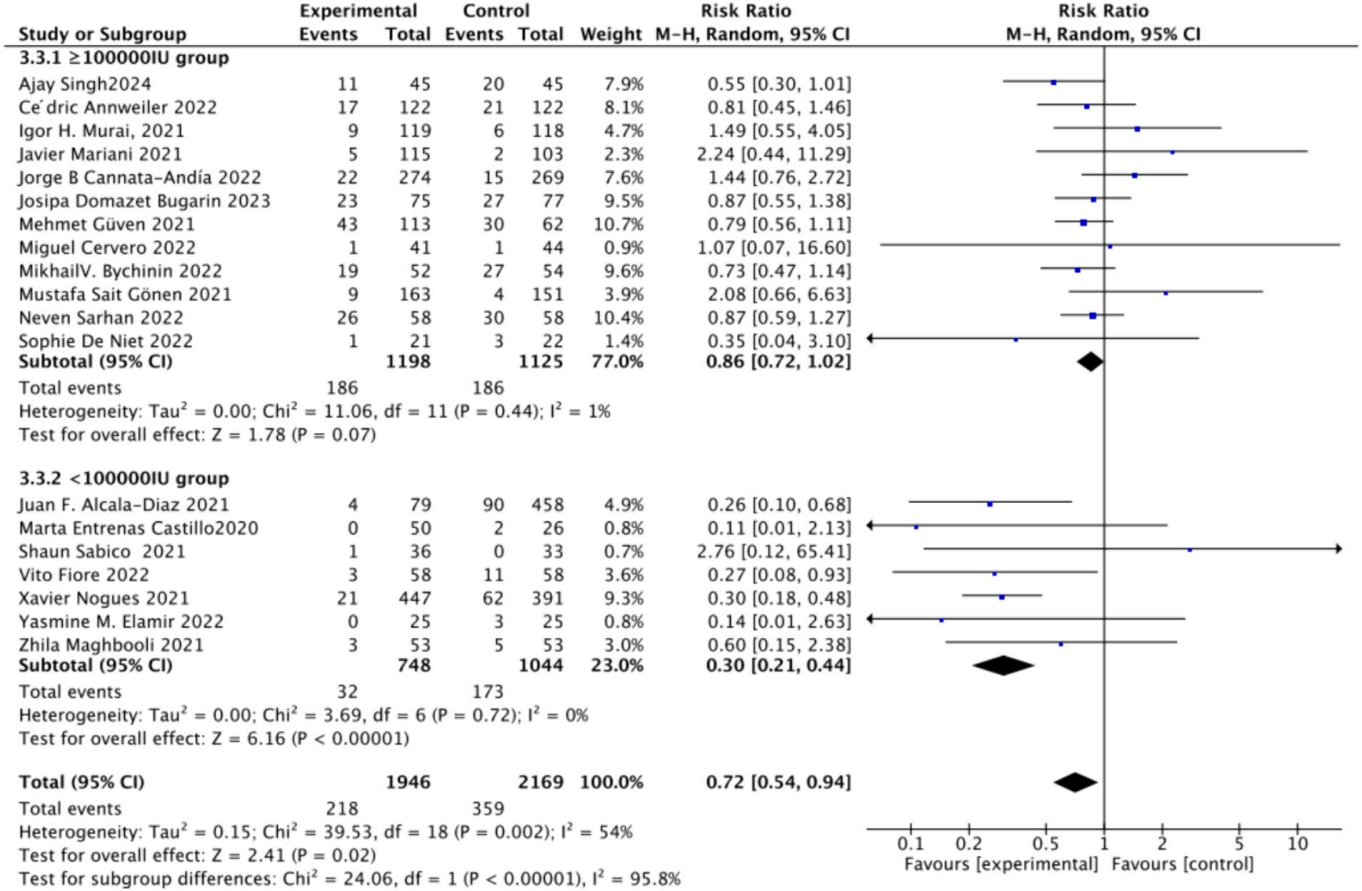
Forest plot of mortality rate by total Vitamin D dosage intake within the first 14 days of hospitalization.

**Figure 6:**
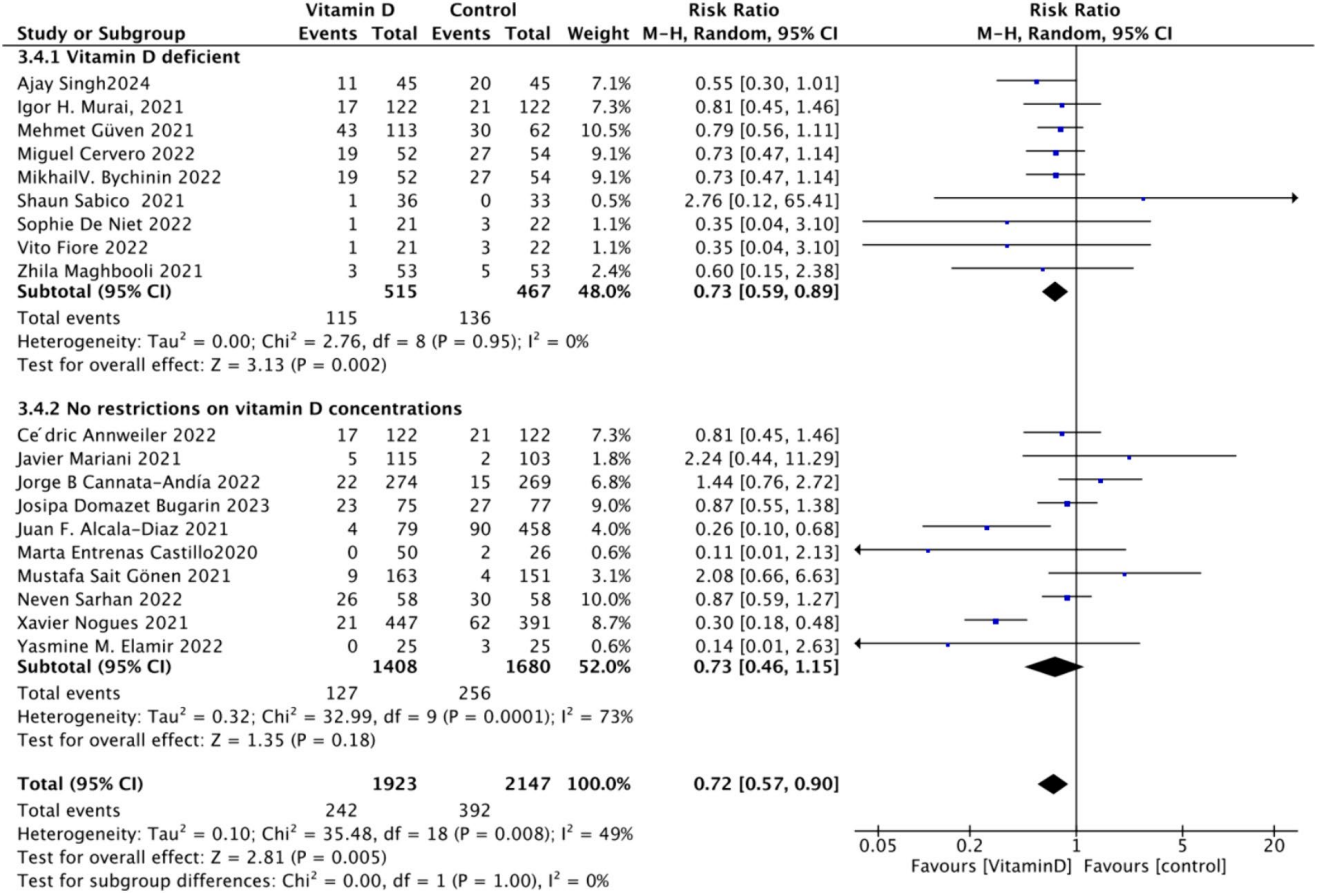
Forest plot of mortality rate by baseline serum Vitamin D concentration restrictions among study populations.

- To analyze the mortality rate, we included 19 studies with a total of 4,115 participants. We first conducted a heterogeneity test, which revealed an *I*^2^ value greater than 50% (*I*^2^ = 54%, *p* = 0.02). As a result, we employed a random-effects model for statistical analysis. Data analysis showed a total relative risk (RR) of 0.72 (95% CI: 0.54–0.94), with a significance level of *p* = 0.02 (Figure 3).
- Subgrouped by administration method: For the subgroup analysis based on the mortality rate, we categorized the intervention groups into two subgroups according to the Vitamin D intake method: single dose or continuous dose. Studies administering Vitamin D only once upon admission were classified as the single dose group (dose frequency = 1), while those administering multiple doses after admission were classified as the continuous dose group (dose frequency ≧ 2). Seven studies employed a single-dose regimen, while 12 studies used a continuous-dose regimen. The combined relative risk (RR) for the single-dose subgroup of the intervention group was 0.88 (95% CI: 0.69–1.12), with an *I*^2^ value of 21% and *p* = 0.3, indicating no statistically significant reduction in mortality. Conversely, the RR for the continuous-dose subgroup was 0.53 (95% CI: 0.34–0.83), with an *I*^2^ value of 55% and *p* = 0.006, showing a statistically significant reduction in mortality (Figure 4).
- Subgrouped by dosage: For the second subgroup analysis based on mortality rate, we categorized the intervention groups according to the total Vitamin D intake. This was calculated according to the total dosage during the first 14 days of hospitalization. There were two groups: ≥100,000 International Units (IU and <100,000 IU). Of the selected studies, 12 administered total doses of ≥100,000 IU of Vitamin D in the intervention group, while seven administered <100,000 IU. The combined RR value for the ≥100,000 IU intake group was 0.85 (95% CI: 0.71–1.02), with an *I*^2^ value of 1% and *p* = 0.07, indicating no statistically significant reduction in mortality. In contrast, the RR for the <100,000 IU intake group was 0.30 (95% CI: 0.21–0.44), with an *I*^2^ value of 0% and *p* < 0.0001, showing a statistically significant reduction in mortality (Figure 5).
- Subgrouped by serum Vitamin D concentrations upon admission: For the third subgroup analysis based on mortality rate, we classified the studies according to the serum Vitamin D concentration restrictions applied to the included patients. The groups were divided into those with Vitamin D deficiency (serum 25 (OH)D (25-Hydroxyvitamin D) < 30 ng/mL) and those with no restrictions on serum Vitamin D concentrations. Of the selected studies, 9 limited inclusion to patients with serum 25 (OH) D < 30 ng/mL, while the other 10 studies had no restrictions on serum 25 (OH) D levels. The combined risk ratio (RR) for the group with serum 25 (OH) D < 30 ng/mL was 0.73 (95% CI: 0.59–0.89), with an *I*^2^ value of 0% and *p* = 0.002, indicating a statistically significant reduction in mortality. In contrast, the RR for the group with no restrictions on serum 25 (OH) D concentrations was 0.73 (95% CI: 0.46–1.15), with an *I*^2^ value of 73% and *p* = 0.18, suggesting no statistically significant reduction in mortality (Figure 6).

#### 3.4.2 Effect of oral vitamin D dosage on the risk of ICU admission

**Figure 7:**
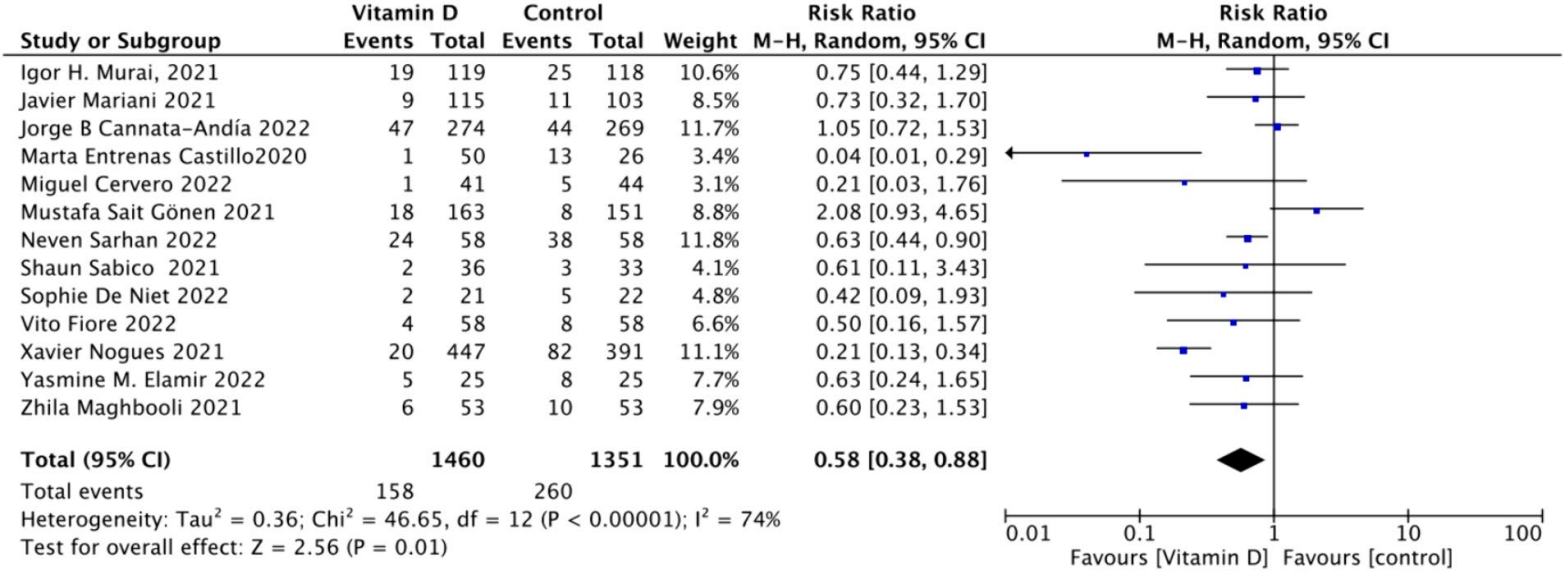
Forest plot of ICU admission rate.

**Figure 8:**
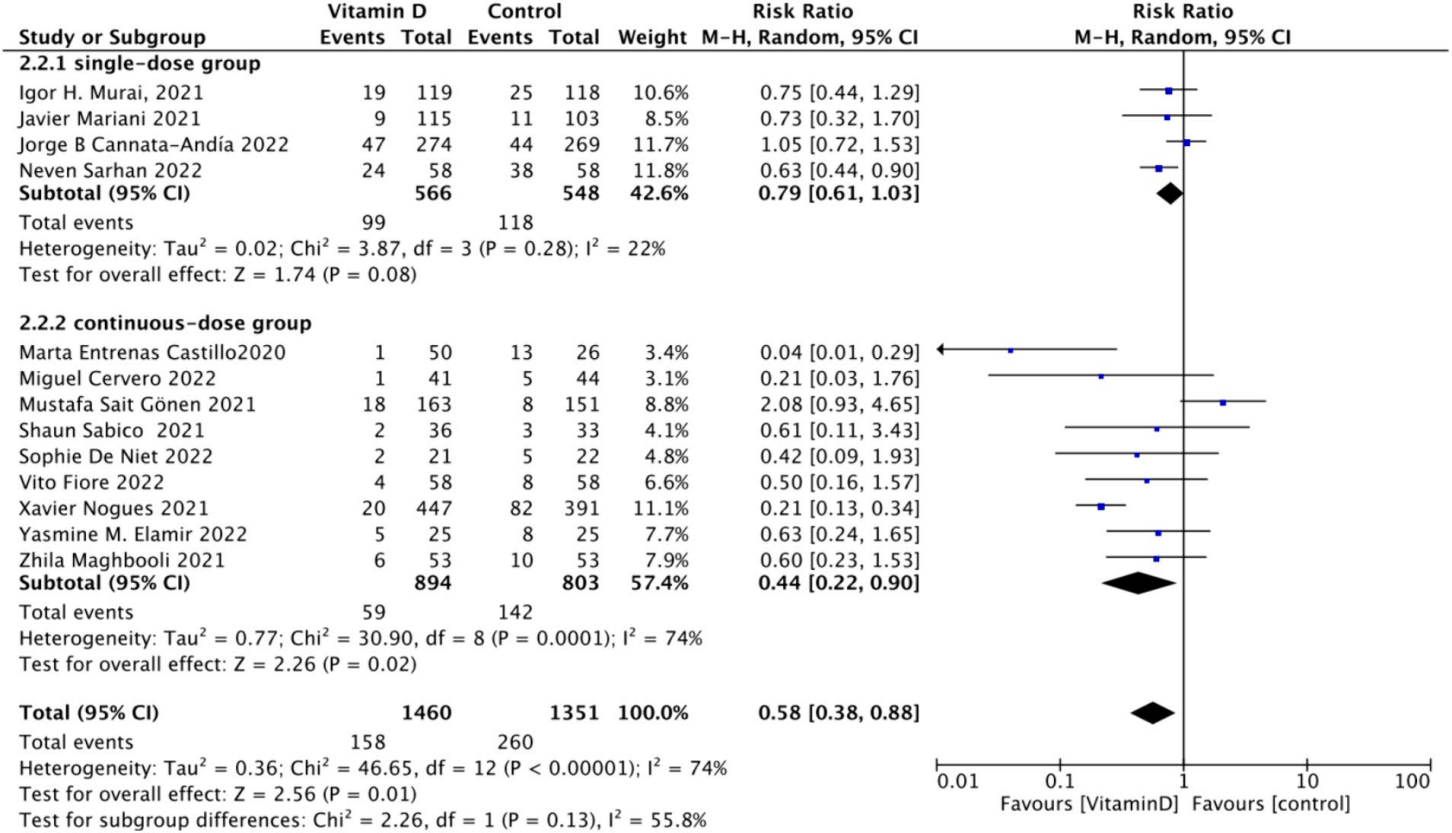
Forest plot of ICU admission rate subgrouped by administration method.

**Figure 9:**
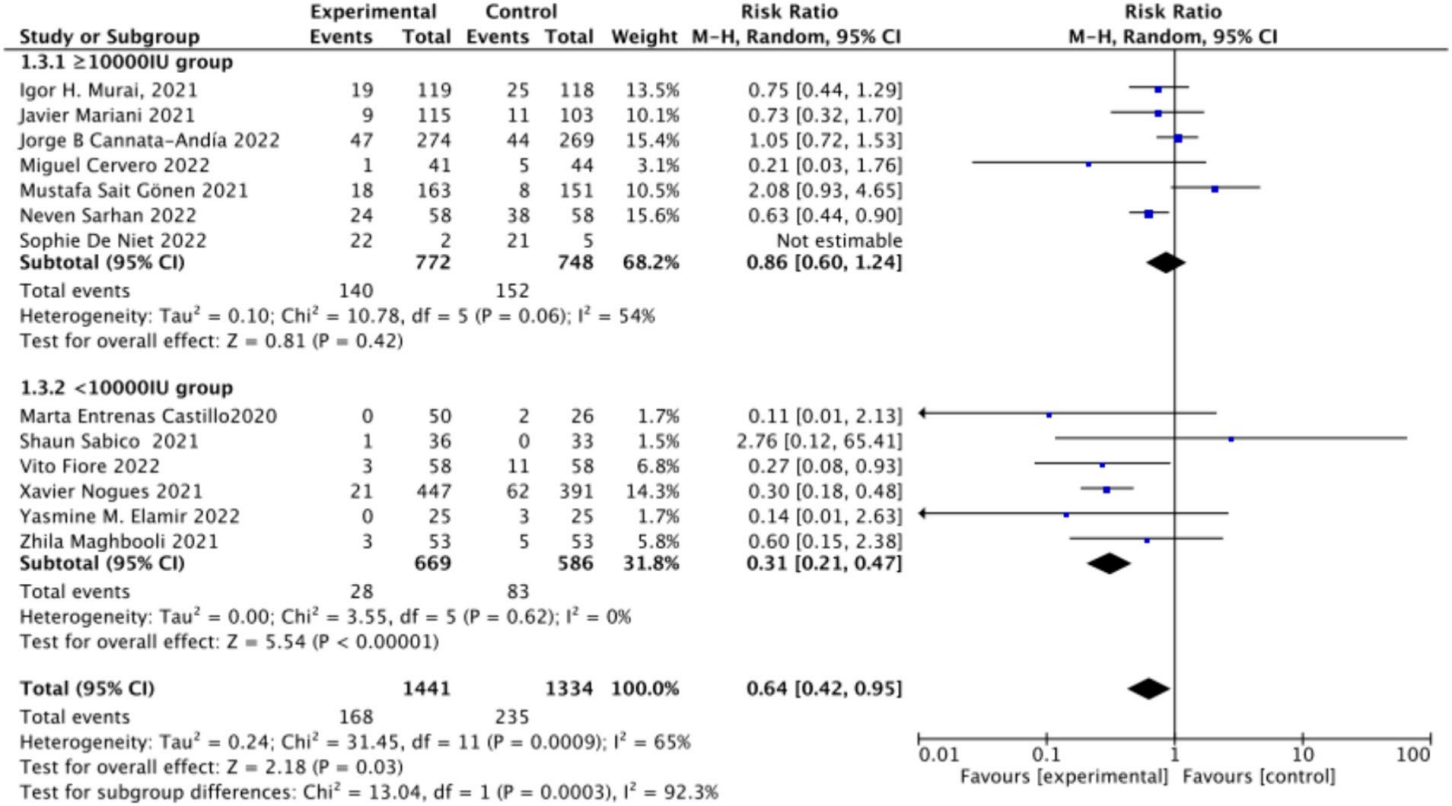
Forest plot of ICU admission subgrouped by dosage.

**Figure 10:**
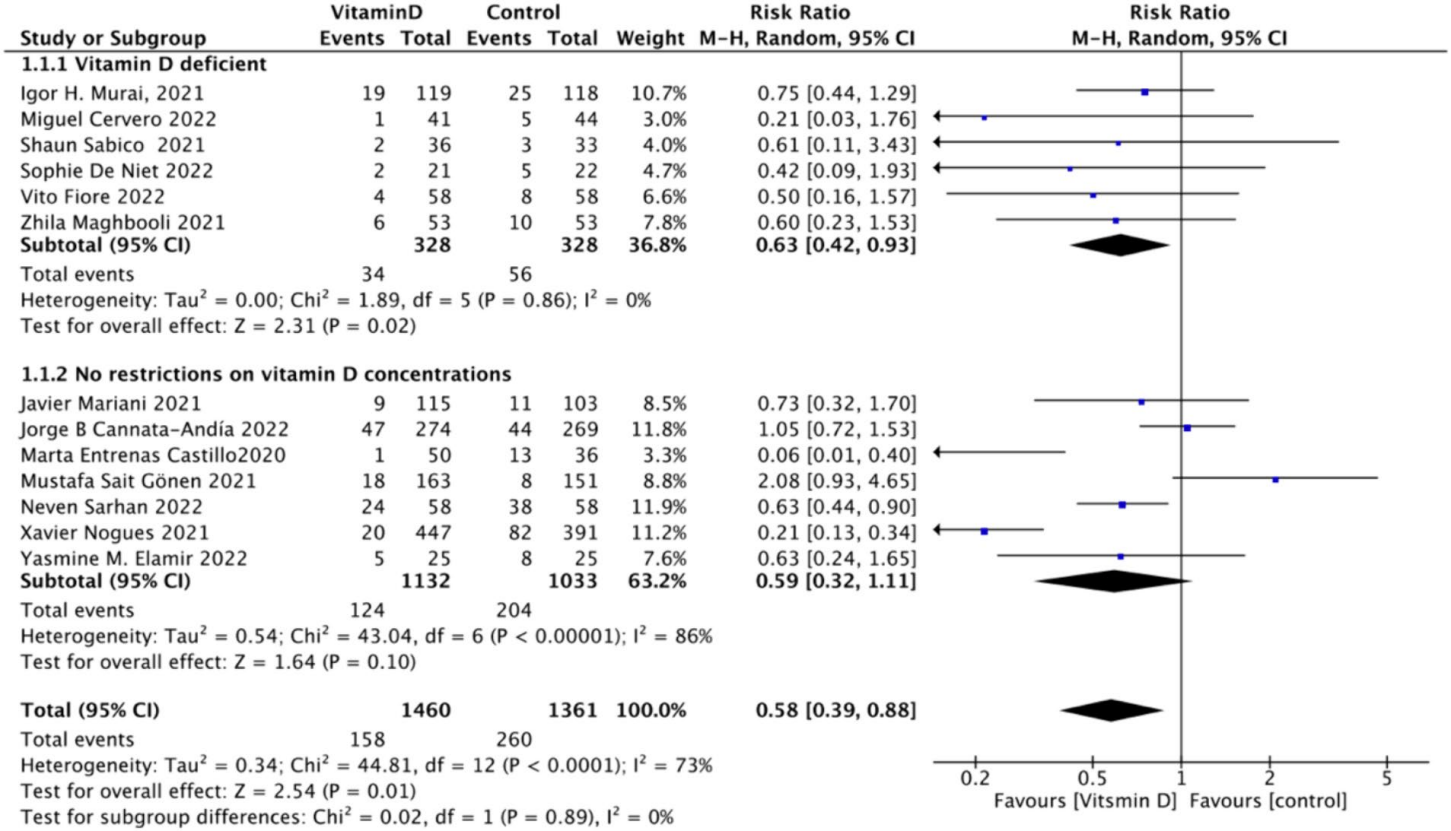
Forest plot of ICU admission by baseline serum Vitamin D concentration restrictions among study populations.

- When assessing ICU admission rate as an outcome, a total of 13 studies involving 2,811 participants were included. The pooled relative risk (RR) was 0.58 (95% CI: 0.38–0.88), with significant heterogeneity (*I*^2^ = 74%, *p* = 0.01) (Figure 7).
- Subgrouped by administration method: Of the included studies, four applied single-dose administration, while nine used continuous administration. The combined RR value for the single-dose Vitamin D subgroup was 0.79 (95% CI: 0.61–1.03), with low heterogeneity (*I*^2^ = 22%, *p* = 0.08), indicating no statistically significant reduction in ICU admission rates. In contrast, the combined RR for the continuous administration subgroup was 0.44 (95% CI: 0.22–0.90), with substantial heterogeneity (*I*^2^ = 74%, *p* = 0.02), showing a statistically significant reduction in ICU admission rates (Figure 8).
- Subgrouped by dosage: The studies were further subdivided according to the Vitamin D dosage. Seven studies utilized Vitamin D dosages of ≥100,000 IU, while six offered dosages of <100,000 IU. The combined RR value for the ≥100,000 IU subgroup over 14 days was 0.86 (95% CI: 0.6–1.24), with moderate heterogeneity (*I*^2^ = 54%, *p* = 0.42), indicating no statistically significant reduction in ICU admission rates. However, the combined RR for the <100,000 IU subgroup was 0.31 (95% CI: 0.21–0.47), exhibiting low heterogeneity (*I*^2^ = 0%, *p* = 0.001), showing a statistically significant reduction in ICU admission rates (Figure 9).
- Subgrouped by serum Vitamin D concentrations upon admission: Among the selected studies, 6 restricted inclusion to patients with serum 25 (OH) D levels <30 ng/mL, while the remaining 7 studies imposed no restrictions on serum 25 (OH) D levels. The pooled risk ratio (RR) for the group with serum 25 (OH) D < 30 ng/mL was 0.63 (95% CI: 0.42–0.93), *I*^2^ = 0%, and *p* = 0.02, indicating a statistically significant reduction in ICU admission. Conversely, for the group with no restrictions on serum 25 (OH) D concentrations, the RR was 0.59 (95% CI: 0.32–1.11), (*I*^2^ = 86%) and *p* = 0.1, indicating no statistically significant reduction in ICU admission (Figure 10).

#### 3.4.3 Effect of oral vitamin D on hospitalization duration

**Figure 11:**
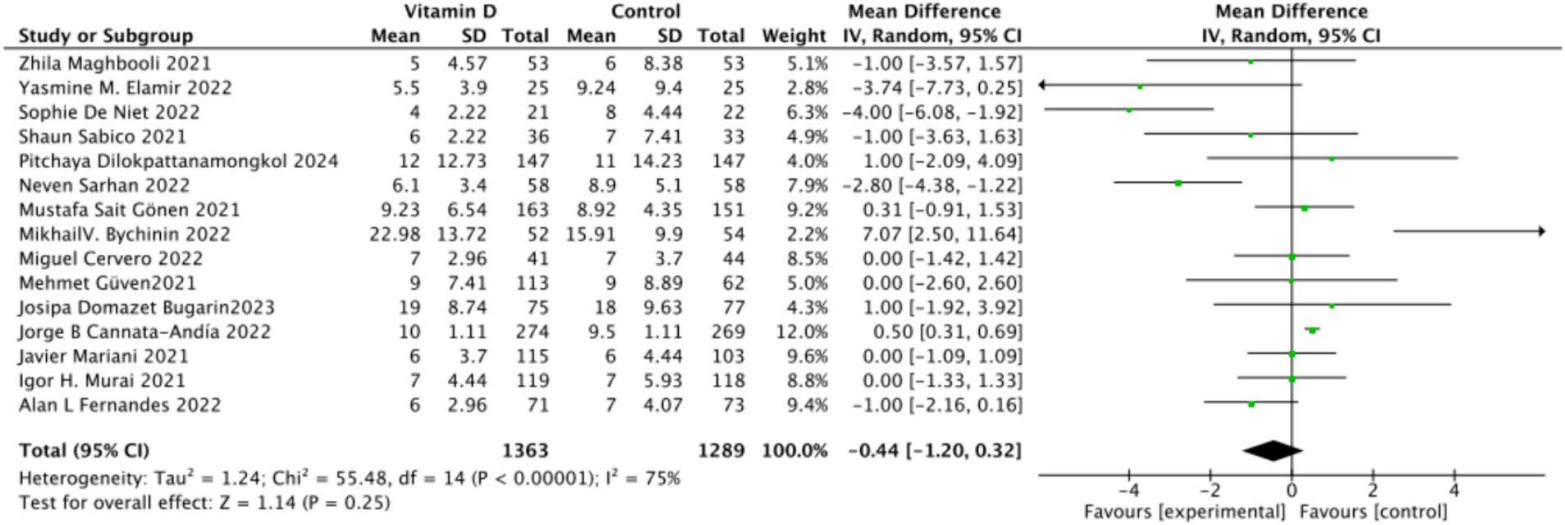
Forest plot of hospital stay duration.

**Figure 12:**
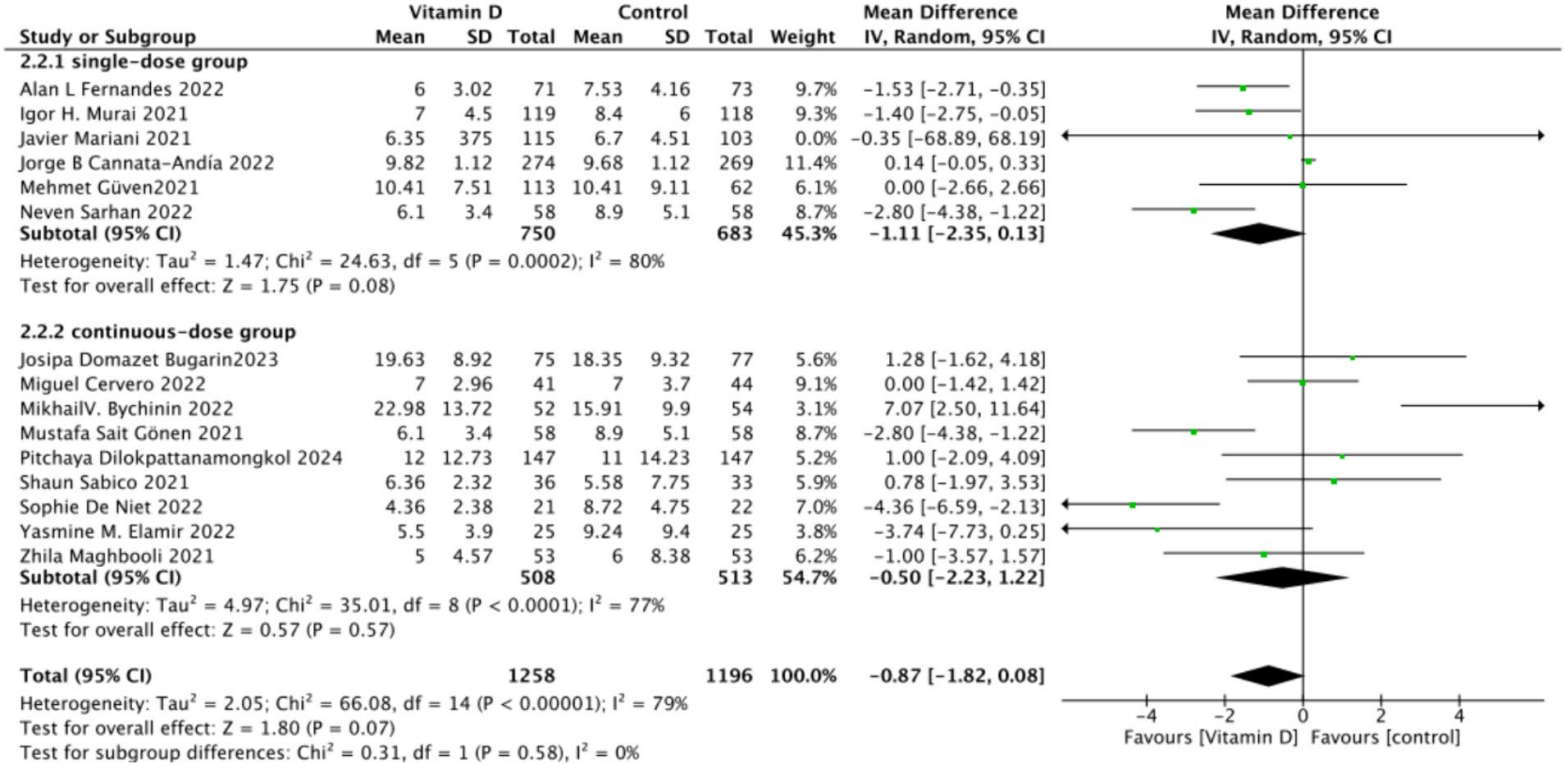
Forest plot of hospital stay duration by subgroup according to administration method.

**Figure 13:**
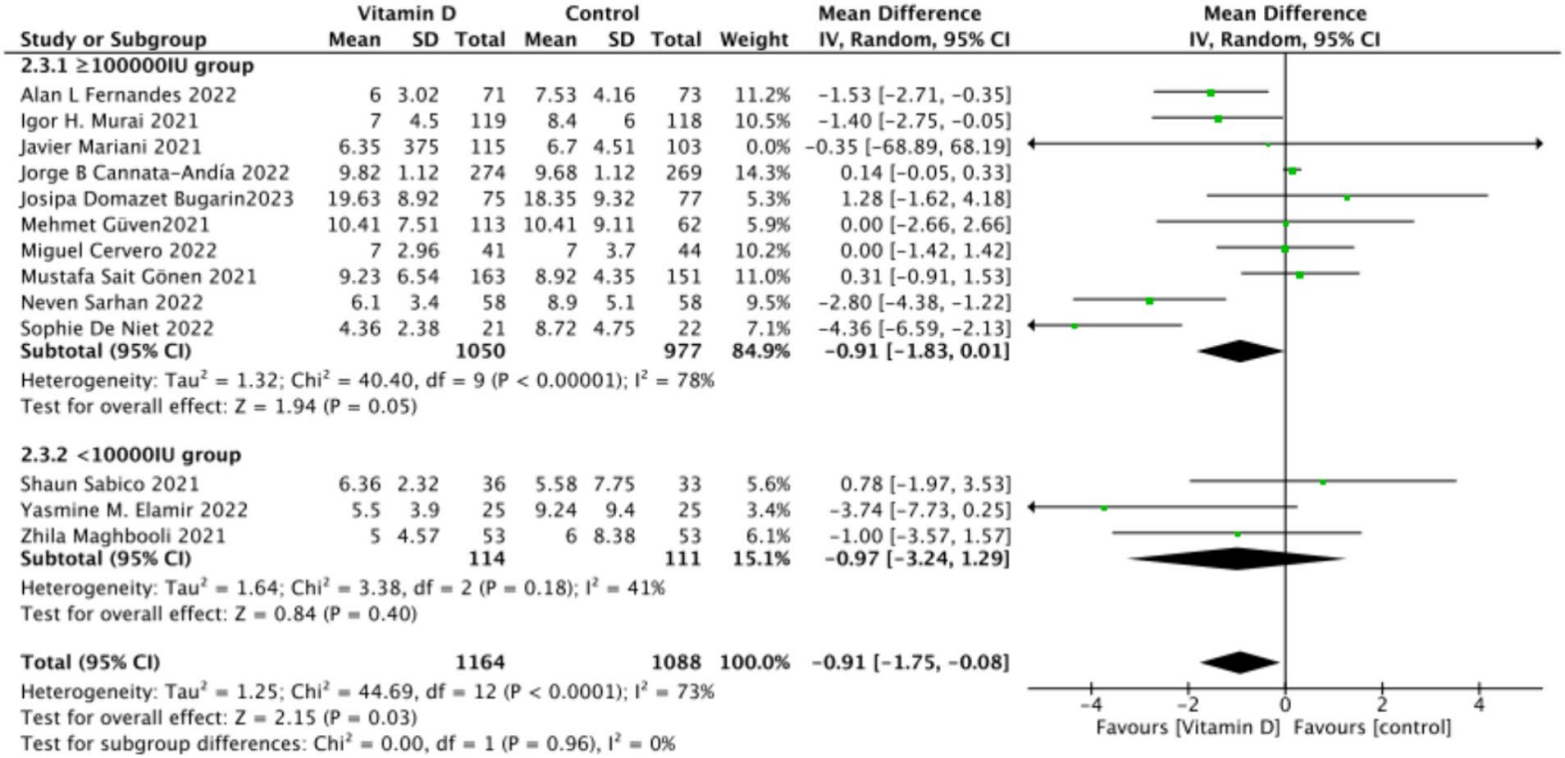
Forest plot of hospital stay duration subgrouped by total Vitamin D dosage administered within 14 days of admission.

**Figure 14:**
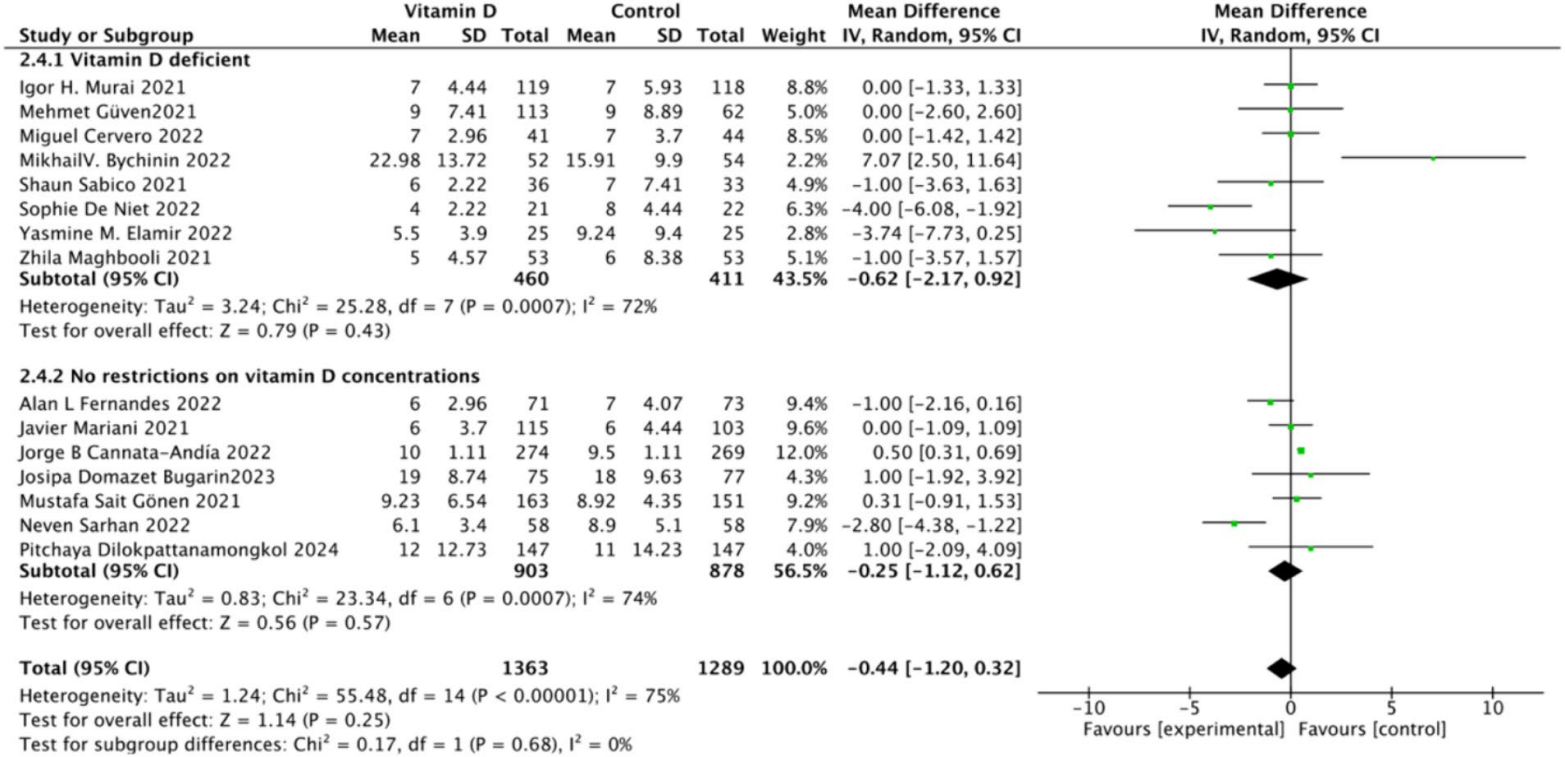
Forest plot of hospital stay duration subgrouped by baseline serum Vitamin D concentration.

- There were 15 studies involving 2,652 participants that assessed hospitalization duration as an outcome. The pooled results showed no significant difference between the Vitamin D group and the control group, with a standardized mean difference (MD) of −1 (95% CI: −2.16 to 0.16; *p* = 0.13) (Figure 11).
- Subgrouped by administration method: Six studies administered Vitamin D in single doses, while nine studies offered them continuously. There was a negligible difference between the single-dose subgroup and the control group, with an MD of −1.11 (95% CI: −2.35 to 0.13; *p* = 0.08). Similarly, there was no significant difference between the subgroup receiving continuous Vitamin D administration and the control group, with an MD of −0.50 (95% CI: −2.23 to 1.22; *p* = 0.57) (Figure 12).
- Subgrouped by dosage: Ten studies administered Vitamin D dosages of ≥100,000 IU, while three studies gave <100,000 IU. There was no significant difference between the subgroup receiving dosages of ≥100,000 IU and the control group, with an MD of −0.91 (95% CI: −1.83 to 0.01; *p* = 0.05). Similarly, there was an insignificant difference between the subgroup receiving <100,000 IU and the control group, with an MD of −0.97 (95% CI, −3.24 to 1.29; *p* = 0.4) (Figure 13).
- Subgrouped by serum Vitamin D concentrations upon admission: Among the selected studies, 8 restricted inclusion to patients with serum 25(OH) D levels <30 ng/mL, while the remaining 7 studies imposed no restrictions on serum 25(OH)D levels, to evaluate the impact on the length of hospital stay. The pooled mean difference for the group with serum 25(OH)D < 30 ng/mL was −0.62 (95% CI: −2.17 to 0.92), with an *I*^2^ of 72% and *p* = 0.43. For the group with no restrictions on serum 25(OH)D concentrations, the mean difference was −0.25 (95% CI: −1.12 to 0.62), with an *I*^2^ of 75% and *p* = 0.57 (Figure 14).

#### 3.4.4 Impact of oral vitamin D on endotracheal intubation rate

**Figure 15:**
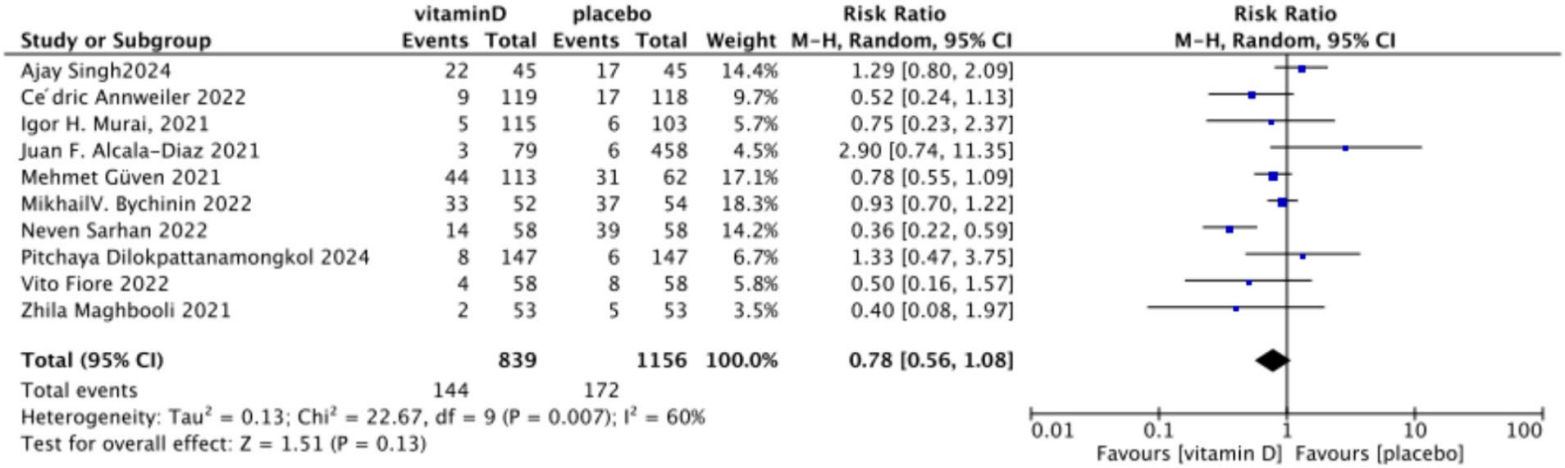
Forest plot of tracheal intubation rate.

**Figure 16:**
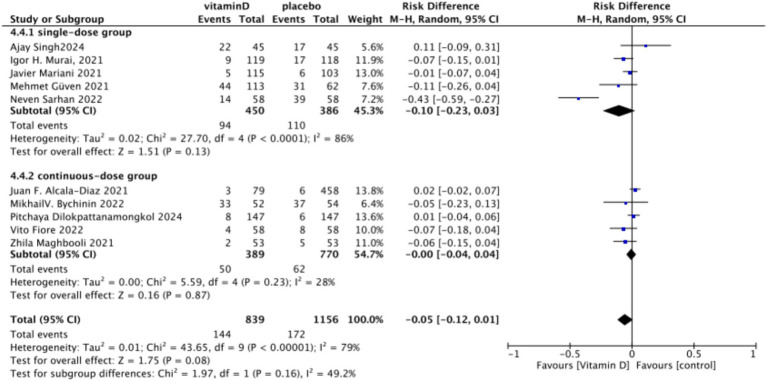
Forest plot of tracheal intubation rate subgrouped by administration method.

**Figure 17:**
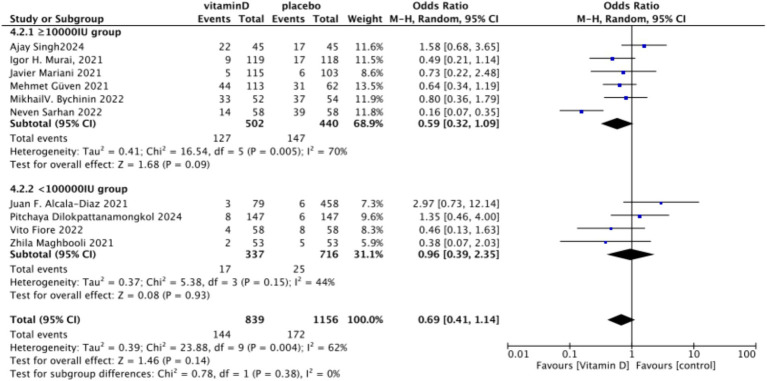
Forest plot of tracheal intubation rate by subgroup according to total Vitamin D dose administered within 14 days of admission.

**Figure 18:**
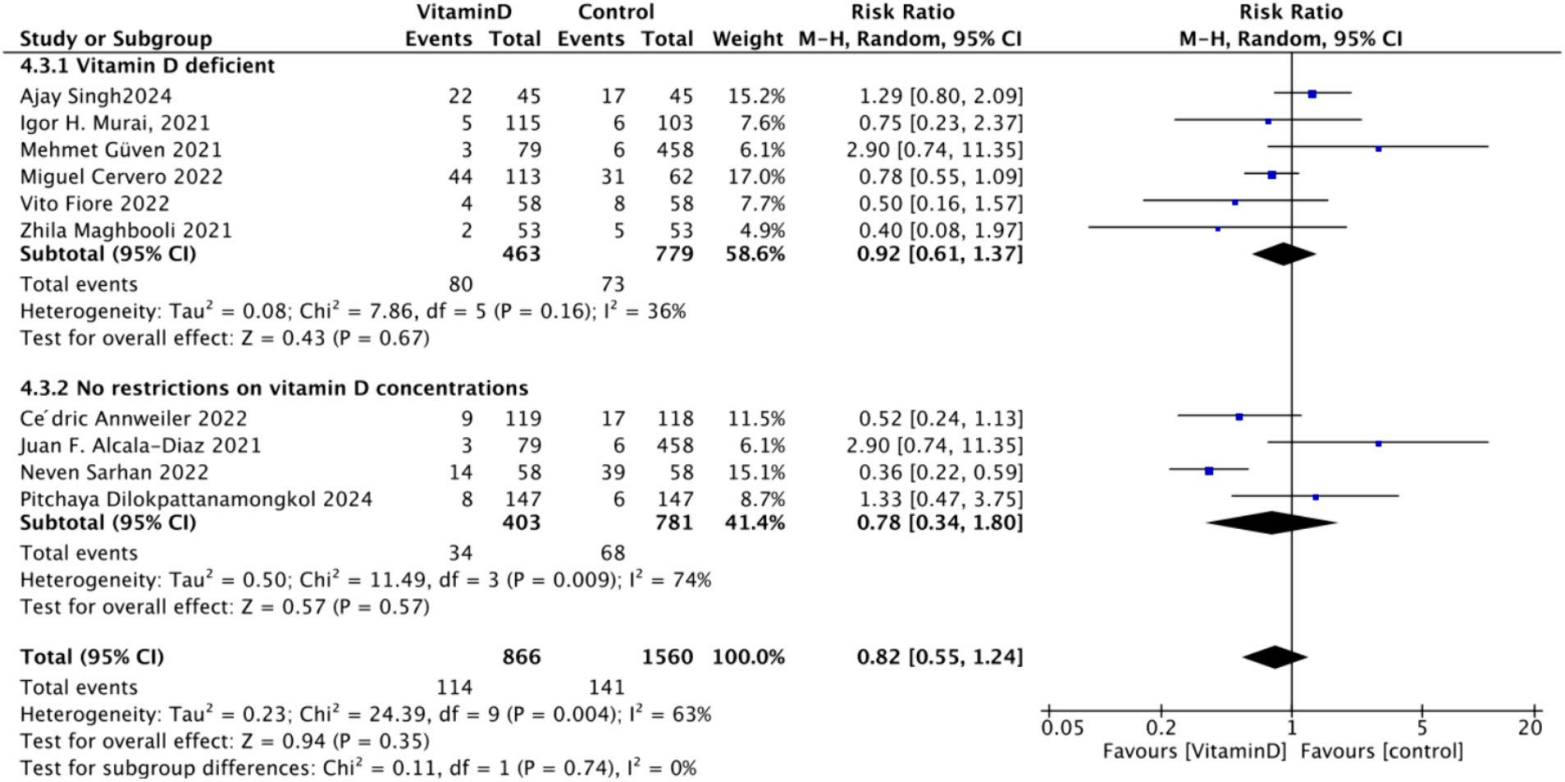
Forest plot of tracheal intubation rate by subgroup according to baseline serum Vitamin D concentrations.

- In the analysis of endotracheal intubation, 10 studies involving 1,995 participants were included. The results indicated no significant difference between the Vitamin D group and the control group, with an RR value of 0.78 (95% CI: 0.56, 1.08; *p* = 0.13) (Figure 15).
- Subgrouped by administration method: Five studies involved one-time doses and five studies utilized continuous dosing. There was a negligible difference between the intervention group with one-time Vitamin D dosage and the control group. Specifically, the RR value was −0.10 (95% CI, −0.23, 0.03; *p* = 0.13). Similarly, no significant difference was observed between the intervention group with continuous Vitamin D dosage and the control group, with an RR value of −0.00 (95% CI: −0.04 to 0.04; *p* = 0.87) (Figure 16).
- Subgrouped by dosage: Six studies included intervention groups with Vitamin D dosages ≥100,000 IU, while four studies involved groups with dosages <100,000 IU. There were no significant differences between the intervention group with dosages of ≥100,000 IU and the control group. The RR value was 0.59 (95% CI: 0.32, 1.09; *p* = 0.09). Equally, no noticeable differences were observed between the intervention group with Vitamin D dosages of <100,000 IU and the control group, with an RR value of 0.96 (95% CI: 0.39–2.35; *p* = 0.93) (Figure 17).
- Subgrouped by serum Vitamin D concentrations upon admission: Among the selected studies, 6 restricted inclusion to patients with serum 25(OH)D levels <30 ng/mL, while the remaining 4 studies imposed no restrictions on serum 25(OH)D levels, to evaluate the impact on the of intubation. The pooled risk ratio (RR) for the group with serum 25(OH)D < 30 ng/mL was 0.92 (95% CI: 0.61 to 1.37), with an *I*^2^ of 36% and *p* = 0.67. For the group with no restrictions on serum 25(OH)D concentrations, the RR was 0.78 (95% CI: 0.34 to 1.80), with an *I*^2^ of 74% and *p* = 0.57 (Figure 18).

### 3.5 Administration method

Continuous, multiple-dose administration resulted in lower heterogeneity (*I*^2^ = 55% for mortality, *I*^2^ = 74% for ICU admissions) and significant reductions in both mortality (*p* = 0.006) and ICU admissions (*p* = 0.02). Single-dose administration, which showed higher heterogeneity and no significant improvements, underscores that continuous dosing provides more consistent and effective results.

Total Dosage Over 14 Days: Lower dosages (<100,000 IU) led to reduced heterogeneity (*I*^2^ = 0% for both mortality and ICU admissions) and significant outcome improvements (*p* < 0.0001 for mortality, *p* = 0.001 for ICU admissions). Higher dosages (≥100,000 IU) showed moderate heterogeneity and did not achieve similar benefits, indicating that moderate dosing not only improves outcomes but also provides more consistent results.

Baseline Vitamin D Status: Patients with Vitamin D deficiency (25OHD < 30 ng/mL) showed low heterogeneity and significant benefits from supplementation, including reduced mortality (*I*^2^ = 0%, *p* = 0.002) and ICU admission rates (*I*^2^ = 0%, *p* = 0.02). In contrast, high heterogeneity in the no-restriction group (*I*^2^ = 73% for mortality, *I*^2^ = 86% for ICU admission) with no significant benefits suggests that baseline deficiency is crucial for achieving effective outcomes (Table 2).

**Table 2:** Summary table of subgroup analysis.

		Mortality			ICU admission			Length of hospital stay			Endotracheal intubation		
Subgroup criteria	Analysis type	RR (95% CI)	*I*^2^	*p* value	RR (95% CI)	*I*^2^	*p* value	MD (95% CI)	*I*^2^	*p* value	RR (95% CI)	*I*^2^	*p* value
	Total analysis	0.72 (0.54–0.94)	54%	0.02	0.58 (0.38–0.88)	74%	0.01	−1.00 (−2.16 to 0.16)	63%	0.13	0.78 (0.56–1.08)	60%	0.13
Subgroup by administration method	Single-dose	0.88 (0.69–1.12)	21%	0.3	0.79 (0.61–1.03)	22%	0.08	−1.11 (−2.35 to 0.13)	53%	0.08	−0.10 (−0.23 to 0.03)	42%	0.13
	Continuous dose	0.53 (0.34–0.83)	55%	0.006	0.44 (0.22–0.90)	74%	0.02	−0.50 (−2.23 to 1.22)	69%	0.57	−0.00 (−0.04 to 0.04)	45%	0.87
Subgroup by dosage	≥100,000 IU	0.85 (0.71–1.02)	1%	0.07	0.86 (0.60–1.24)	54%	0.42	−0.91 (−1.83 to 0.01)	57%	0.05	0.59 (0.32–1.09)	38%	0.09
	<100,000 IU	0.30 (0.21–0.44)	0%	<0.0001	0.31 (0.21–0.47)	0%	0.001	−0.97 (−3.24 to 1.29)	48%	0.4	0.96 (0.39–2.35)	34%	0.93
Subgroup by baseline vitamin D status	Vitamin D deficient (25OHD < 30 ng/mL)	0.73 (0.59–0.89)	0%	0.002	0.63 (0.42–0.93)	0%	0.02	−0.62 (−2.17 to 0.92)	72%	0.43	0.92 (0.61–1.37)	36%	0.67
	No baseline restriction	0.73 (0.46–1.15)	73%	0.18	0.59 (0.32–1.11)	86%	0.1	−0.25 (−1.12 to 0.62)	75%	0.57	0.78 (0.34–1.80)	74%	0.57

### 3.6 Bias analysis

**Figure 19:**
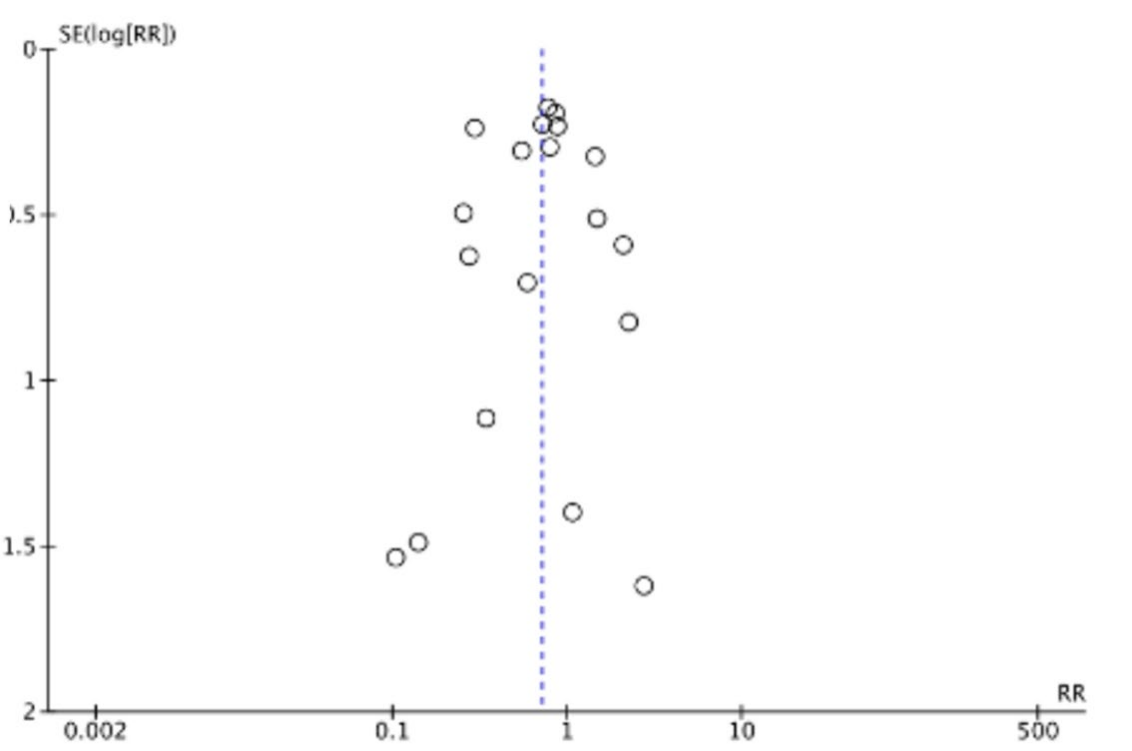
Funnel plot of mortality rate.

**Figure 20:**
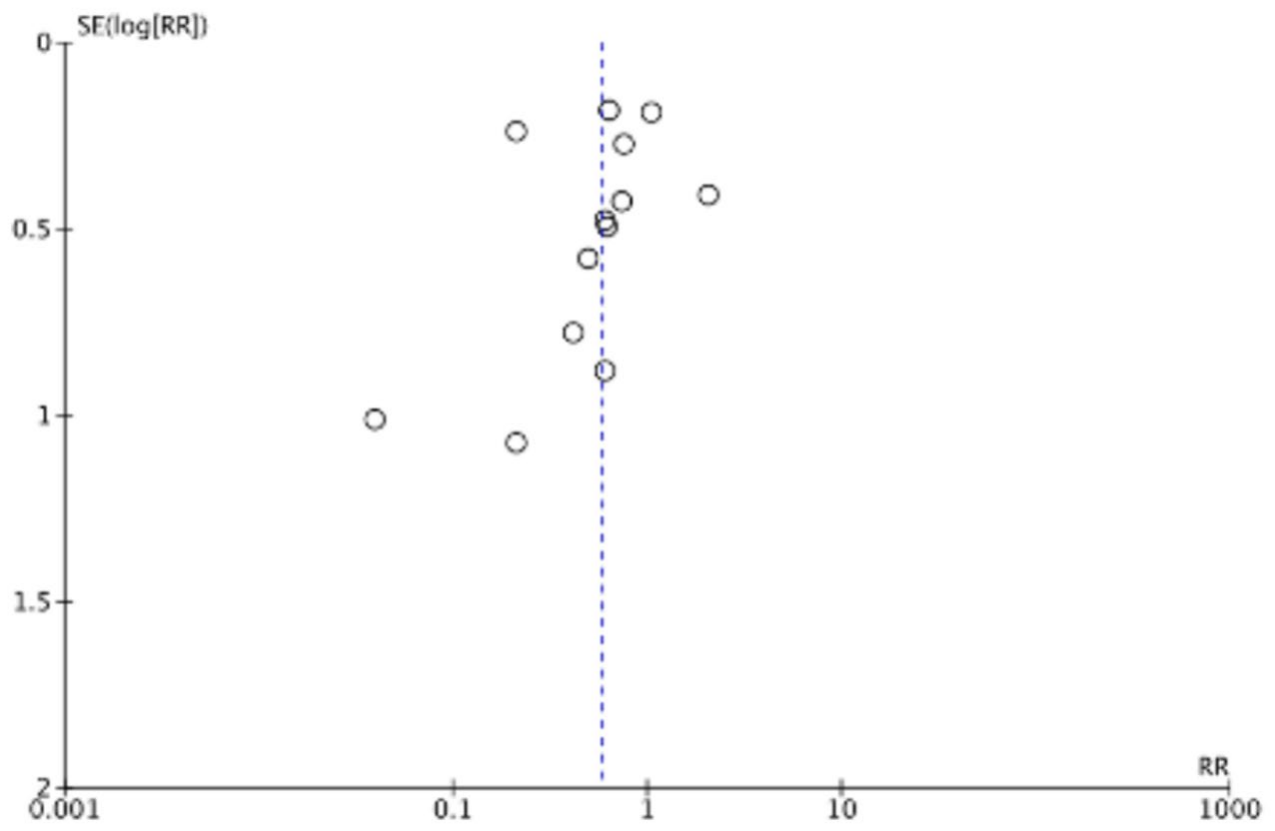
Funnel plot of ICU admission rate.

**Figure 21:**
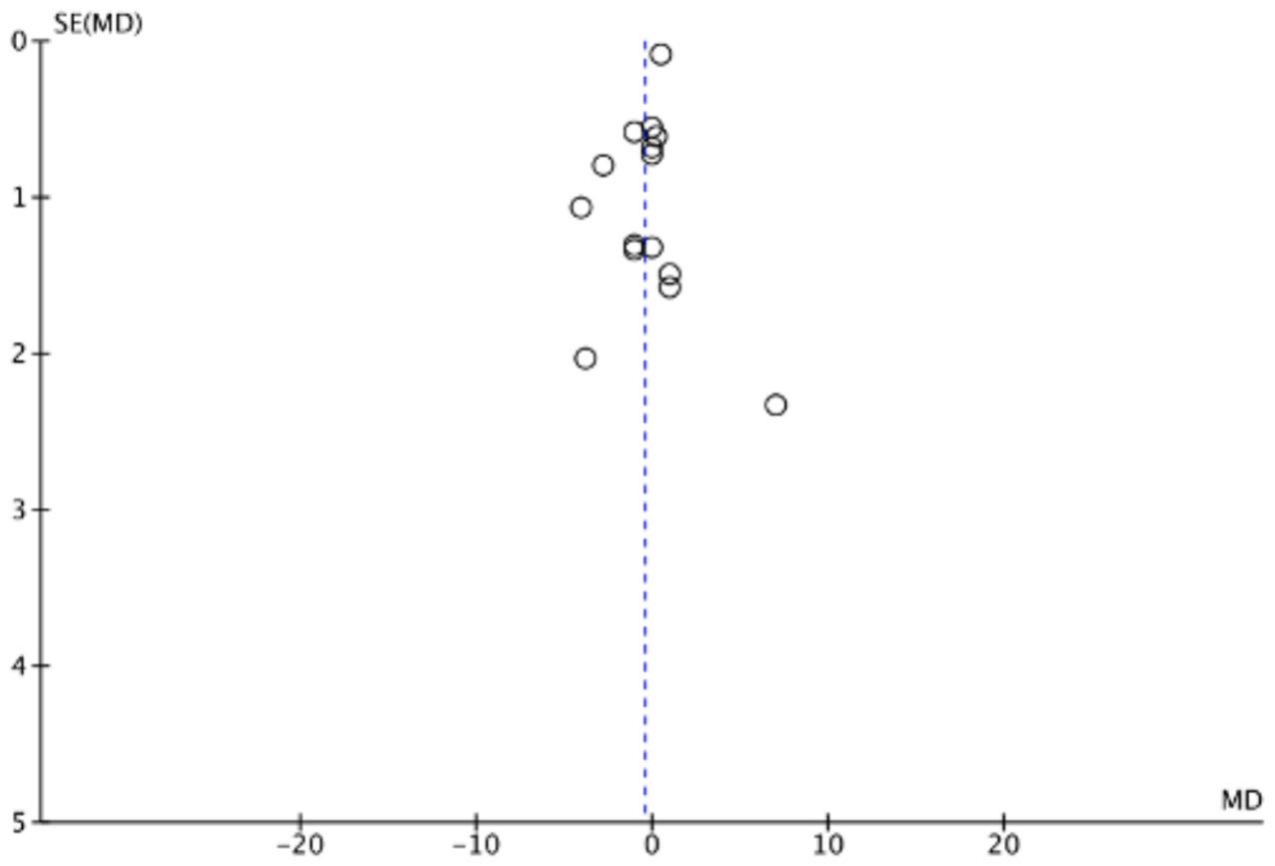
Funnel plot of hospital stay duration.

**Figure 22:**
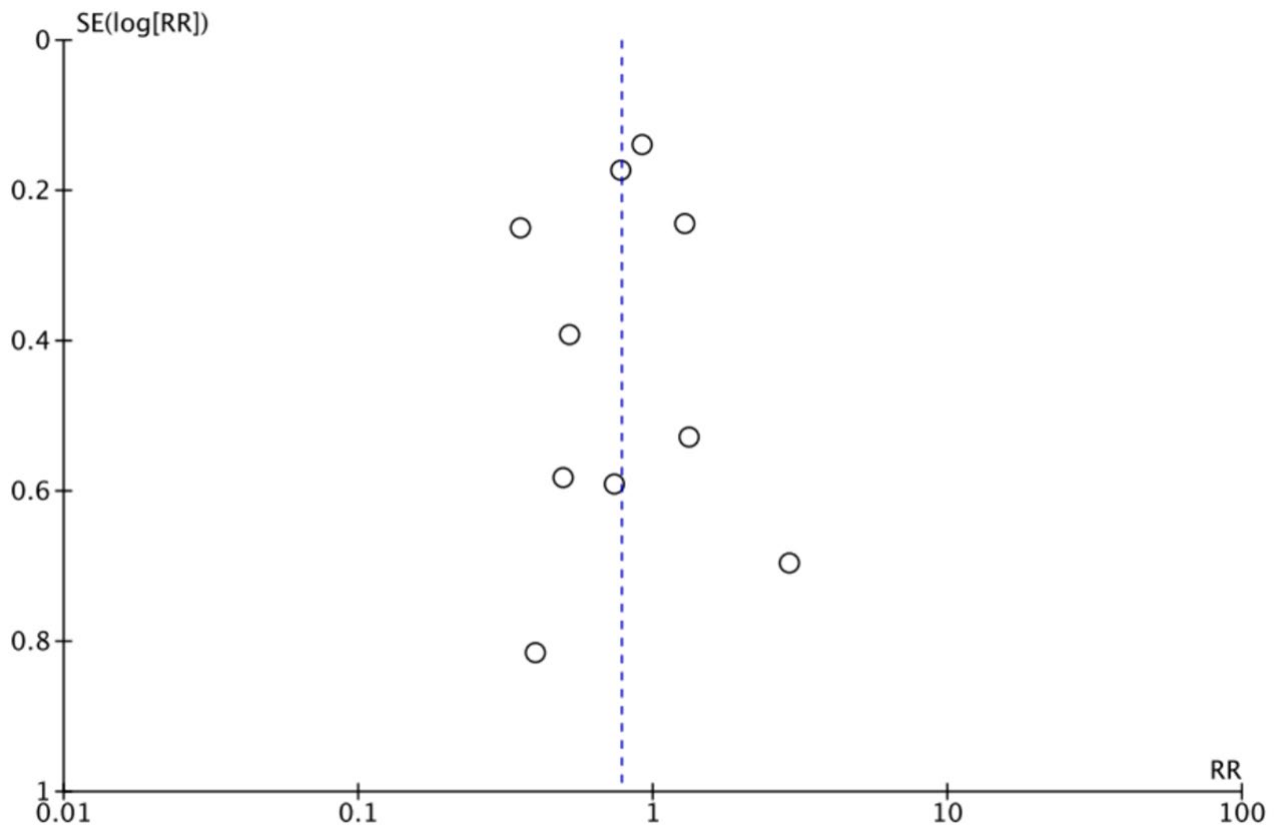
Funnel plot of tracheal intubation rate.

The funnel plots (Figures 19–22) are approximately symmetrical on both sides, indicating the absence of meaningful publication bias. Subsequently, we conducted a sensitivity analysis, systematically assessing the impact of removing individual studies. We established that no single study exerted substantial influence on the overall results, suggesting that our findings were robust.

## 4 Discussion

In this systematic review and meta-analysis, we investigated the impact of oral Vitamin D supplementation on the prognosis of COVID-19 patients across 21 studies involving 4,553 individuals. Our findings suggest that Vitamin D administration following COVID-19 infection leads to improved mortality rates and reduced ICU admission rates. However, we did not observe any significant effects on hospital stay duration or intubation rates.

To achieve the optimal therapeutic effect of Vitamin D supplementation, we conducted various subgroup analyses. Patients were classified based on three main criteria: the mode of Vitamin D administration upon hospital admission (single-dose versus multiple-dose), the total Vitamin D dosage over 14 days (≥100,000 IU versus <100,000 IU), and baseline Vitamin D levels (serum 25(OH)D < 30 ng/mL versus no restriction). Our analysis revealed that continuous, multiple-dose administration and total dosages of <100,000 IU over 14 days were strongly associated with reduced mortality and ICU admission rates. In contrast, single-dose administration and total dosages ≥100,000 IU did not show noticeable improvements in outcomes. Furthermore, patients with baseline Vitamin D deficiency (25OHD < 30 ng/mL) experienced significant reductions in mortality and ICU admission rates following supplementation, while those without baseline restrictions did not show the same level of benefit.

By implementing strategic subgroup analyses, we effectively reduced heterogeneity and achieved more consistent results. For example, grouping patients by total Vitamin D dosage over 14 days and baseline Vitamin D levels significantly lowered heterogeneity. The <100,000 IU total dose subgroup and the Vitamin D deficiency subgroup (25OHD < 30 ng/mL) frequently showed heterogeneity values of zero in mortality and ICU admission outcomes (Table 2). These findings suggest that lower, continuous doses of Vitamin D are more effective than higher, single doses and that supplementation in Vitamin D-deficient populations leads to greater improvements in clinical prognosis. By highlighting the importance of dosing strategies and baseline Vitamin D status, our study provides a potential explanation for the conflicting results observed in previous research on Vitamin D supplementation and COVID-19 outcomes. The inconsistencies in earlier studies may be attributed to differences in patient selection, dosing regimens, and a lack of consideration for baseline Vitamin D status.

Since the outbreak of the novel coronavirus infection in 2019, numerous studies have explored the relationship between Vitamin D supplementation and the prognosis for COVID-19 infection. However, results from clinical trials vary and meta-analyses on this topic also exhibit discrepancies. For instance, the majority of meta-analyses conclude that oral Vitamin D supplementation has a negligible impact on the mortality rate of COVID-19 patients (22, 43–51). Nevertheless, a few reports suggest that supplementation reduces COVID-19 patient mortality rates (52, 53). Similarly, most meta-analyses state that Vitamin D supplementation significantly lowers the ICU admission rate of COVID-19 patients (43, 46, 47, 49–51, 53), although some studies contradict these findings (22, 45). These discrepancies may be due to the Vitamin D supplementation dosage and method, necessitating further clinical trials and meta-analyses for deeper investigation. Our study evaluated the mortality rate, ICU admission rate, length of hospital stay, and intubation rate of hospitalized COVID-19 patients. The positive feature of this study is that we conducted subgroup analyses based on the mode of administration and 14-day total Vitamin D intake, which appreciably reduced the heterogeneity in some subgroups. Additionally, our study yielded new findings regarding total intake, partially clarifying the discrepancies encountered in previous meta-analyses.

Our conclusions regarding the impact of administration methods on the clinical outcomes of COVID-19 patients suggest that continuous administration of Vitamin D is superior to one-time bolus intake. The relationship between Vitamin D supplementation methods and respiratory tract infections has been studied extensively in recent years. Numerous clinical trials have consistently shown that continuous low-dose Vitamin D supplementation is more effective than intermittent high-dose administration. In their respective meta-analyses of numerous randomized controlled trials, Martineau (54) and Jolliffe (6) both concluded that continuous low-dose maintenance Vitamin D supplementation produces a considerably greater protective effect against the risk of acute respiratory tract infections compared to high-dose intermittent dosing. Considering that COVID-19 is a respiratory infection, during the early stages of the COVID-19 pandemic, Griffin (55) recommended continuous administration of Vitamin D for COVID-19 patients based on the relationship between Vitamin D supplementation and respiratory tract infections. Elaborating on this, Feiner Solís et al. (56) conducted a systematic review, summarizing 11 relevant clinical trials. Six of the studies involved continuous Vitamin D administration and five studies involved a single bolus dose. His results revealed that continuous Vitamin D supplementation was associated with better clinical outcomes in patients with COVID-19, whereas a single bolus dose did not improve any clinical outcomes. Our study further corroborates these findings. From the perspective of Vitamin D metabolism, a plausible explanation is that its activation and metabolism are regulated by enzymes. Following a large bolus dose of Vitamin D, the enzyme 24-hydroxylase, which inactivates Vitamin D, may remain active for several weeks as a feedback response (57). This sustained elevation in 24-hydroxylase activity can paradoxically result in intracellular depletion of active Vitamin D, known as the rebound effect, particularly affecting immune cells. In contrast, daily low-dose supplementation maintains consistent Vitamin D activity by preventing the significant upregulation of 24-hydroxylase (58).

To date, there have been no clinical trials or meta-analyses that specifically analyze the effects of total intake within a certain timeframe on the prognosis of COVID-19 patients. In the context of respiratory tract infections and Vitamin D supplementation, the RCT study by Wall-Gremstrup et al. (59) proposed that the probability of respiratory tract infections is significantly higher in the high-dose Vitamin D group (supplemented with 300,000 IU on the first day followed by 1,500 IU/day for the next 150 days) than the non-supplemented group. This study supports the notion that high-dose supplementation does not enhance immunity in infected patients and may even impair innate immunity. Vieth (60) provided a possible explanation from a pharmacological mechanism perspective. They suggested that high-dose Vitamin D3 intake leads to an imbalance in Vitamin D regulatory enzymes including CYP27B1 and CYP24A1 (Cytochrome P450 family 24 subfamily B member 1 and cytochrome P450 family 24 subfamily A member 1), resulting in significant fluctuations in serum 25(OH)D3 concentrations. This results in a fall in the levels of active Vitamin D (1,25(OH)2D3), which assists the immune system in combating respiratory tract infections. Therefore, high doses of Vitamin D may impair immune function. Another possible cause is fibroblast growth factor23 (FGF23), which is increased by high doses of oral Vitamin D but not by sustained low doses of Vitamin D of 2,000 IU or less per day. High concentration of FGF23 in turn significantly inhibited the 1α-hydroxylation of 25(OH)D, resulting in reduced Vitamin D intracellular activation of 1,25(OH)2D, thereby attenuating the immune-enhancing effect of Vitamin D (61). Concerning supplementation dosage and the prognosis of COVID-19 patients, Tentolouris et al. (47) conducted a preliminary analysis in their meta-analysis. They performed a subgroup analysis on single high-dose and low-dose Vitamin D supplementation, concluding that low-dose supplementation reduces the mortality rate and ICU admission rate of COVID-19 patients, while high-dose supplementation does not. However, in their classification of high and low doses, they arbitrarily assigned studies with individual doses of 200,000 IU and 400,000 IU to the high-dose group, while the remainders were categorized as low doses. They did not consider whether the low-dose group was administered continuously, nor did they compare the total intake of Vitamin D within a certain timeframe between the single high-dose and single low-dose groups. As a result, this approach failed to exclude the influence of factors such as continuous application or total intake on clinical outcomes. Our study is currently the only meta-analysis that simultaneously includes an analysis of both the method of Vitamin D intake and the total dosage within a specific period. Thus, the conclusions of this meta-analysis may facilitate the determination of more appropriate dosages and methods of Vitamin D supplementation, thereby achieving better clinical outcomes.

Previous studies have indicated that Vitamin D deficiency is associated with an increased risk of COVID-19 infection and poor outcomes (5, 10, 52, 62), Consequently, supplementing Vitamin D in deficient populations is more likely to improve COVID-19 prognosis, a finding supported by recent meta-analyses (63). However, few randomized clinical trials have directly grouped patients based on baseline Vitamin D concentrations. In our study, which included 21 clinical trials, 9 did not specify baseline Vitamin D levels, while the remaining 12 included only Vitamin D-deficient individuals (25OHD < 30 ng/mL). Our subgroup analysis using this criterion found that Vitamin D supplementation significantly improved mortality and ICU admission rates in deficient populations, along with a notable reduction in heterogeneity. In contrast, the group without baseline restrictions showed no significant effect on mortality and a significant effect on ICU admission rates, but with high heterogeneity. This discrepancy may be attributed to differences in baseline Vitamin D concentrations. Therefore, selecting appropriate patient populations for Vitamin D supplementation is crucial for optimizing its therapeutic efficacy.

Due to the significant variations in vitamin D supplementation doses and effects in existing clinical trials, there is currently no clear recommendation or consensus for the use of vitamin D specifically for COVID-19 patients. These studies have not provided explicit guidance on the specific applications and dosages of vitamin D in the treatment of COVID-19. As a result, most recommendations focus primarily on the preventive use of vitamin D against COVID-19, drawing on guidelines from other diseases. The main goal is to enhance immune function by maintaining adequate vitamin D levels. For instance, in 2020, international nutritional guidelines recommended a daily intake of 400 IU of vitamin D as a preventive measure against COVID-19, particularly for individuals with limited sun exposure (64, 65). However, in 2022, Griffin G conducted a critical review of the role of vitamin D in the prevention and treatment of diseases such as rickets, tuberculosis, and respiratory infections. He argued that a daily intake of 400 IU is insufficient to achieve optimal serum vitamin D levels, suggesting an increased daily dosage of 800–1,000 IU. For individuals suspected of having vitamin D deficiency, he recommended a higher initial dose of 4,000 IU per day for the first 4 weeks (55, 66). Our study investigated the effects of vitamin D supplementation in COVID-19 patients, focusing on baseline vitamin D levels, supplementation methods, and dosage. The findings indicate that vitamin D supplementation is significantly more effective in individuals with vitamin D deficiency, with optimal results observed when the total supplementation over 14 days is less than 100,000 IU. Although specific guidelines for vitamin D supplementation during COVID-19 are currently limited, our study is the first to demonstrate that a lower cumulative dose of less than 100,000 IU over 14 days is associated with better outcomes.

This study has certain limitations. Firstly, most of the included trials did not report the baseline blood concentrations of Vitamin D among participants, which limited our ability to accurately compare the supplementation effects between individuals with low and high baseline levels. Moreover, there was considerable variation across studies in terms of Vitamin D dosage, patient populations, and methods of administration. To address these differences and better capture real-world scenarios, we employed a random-effects model. Additionally, differences in baseline health conditions, illness severity, and concurrent medications among participants may have influenced the observed effects of Vitamin D supplementation. As this study did not perform stratified analyses based on these patient characteristics, future research should aim to explore these factors to provide a more comprehensive understanding.

## 5 Conclusion

The findings of this review support the conclusion that Vitamin D supplementation has a positive impact on the clinical outcomes of patients with COVID-19. Our analysis of the mode and dosage of Vitamin D supplementation indicates that continuous intake is associated with greater improvements in COVID-19 patients compared to single-dose treatments. Additionally, a total Vitamin D supplementation of less than 100,000 IU over 14 days is more effective than higher doses of 100,000 IU or more. Furthermore, Vitamin D supplementation shows significantly greater efficacy in individuals with Vitamin D deficiency. To further validate these findings, high-quality, long-term follow-up randomized controlled trials (RCTs) are necessary.

## Data Availability

The authors confirm that the data supporting the findings of this study are available in the article.
