# Supplementary material for: The impact of supplementing vitamin D through different methods on the prognosis of COVID-19 patients: a systematic review and meta-analysis
Source: Front Nutr. 2024 Sep 25;11:1441847. doi: 10.3389/fnut.2024.1441847 (PMC11462671; doi:10.3389/fnut.2024.1441847)
Supplement: Supplementary file 1 [file Data_Sheet_1.PDF]

•

#1	“COVID-19"[Mesh]
#2	(((((((((((((((((((((COVID 19[Title/Abstract]) OR (2019-nCoV Infection[Title/Abstract])) OR (nfection, 2019-nCoV[Title/Abstract])) OR (SARS-CoV-2 Infection[Title/Abstract])) OR (SARS CoV 2 Infection[Title/Abstract])) OR (2019 Novel Coronavirus Disease[Title/Abstract])) OR (2019 Novel Coronavirus Infection[Title/Abstract])) OR (COVID-19 Virus Infections[Title/Abstract])) OR (nfection, COVID-19 Virus[Title/Abstract])) OR (Virus Infection, COVID-19[Title/Abstract])) OR (Coronavirus Disease 2019[Title/Abstract])) OR (Disease 2019, Coronavirus[Title/Abstract])) OR (Coronavirus Disease 19[Title/Abstract])) OR (Severe Acute Respiratory Syndrome Coronavirus 2 Infection[Title/Abstract])) OR (COVID-19 Virus Disease[Title/Abstract])) OR (Disease, COVID-19 Virus[Title/Abstract])) OR (Virus Disease, COVID-19[Title/Abstract])) OR (SARS Coronavirus 2 Infection[Title/Abstract])) OR (2019 nCoV Disease[Title/Abstract])) OR (Disease, 2019-nCoV[Title/Abstract])) OR (COVID-19 Pandemic[Title/Abstract])) OR (Pandemic, COVID-19[Title/Abstract])
#3	#1 OR #2
#4	"Vitamin D"[Mesh]
#5	"Calcitriol"[Mesh]
#6	(((((((((((((((((((((((((vitamin D[Title/Abstract]) OR (calciferol[Title/Abstract])) OR (1 alpha,25-Dihydroxycholecalciferol[Title/Abstract])) OR (1, 25-(OH)2D3[Title/Abstract])) OR (1,25-Dihydroxycholecalciferol[Title/Abstract])) OR (1,25-Dihydroxyvitamin D3[Title/Abstract])) OR (1 alpha,25-Dihydroxyvitamin D3[Title/Abstract])) OR (D3, 1 alpha,25-Dihydroxyvitamin[Title/Abstract])) OR (Bocatrio[Title/Abstract])) OR (Calcijex[Title/Abstract])) OR (Calcitriol KyraMed[Title/Abstract])) OR (KyraMed, Calcitriol[Title/Abstract])) OR (Calcitriol-Nefro[Title/Abstract])) OR (Decostriol[Title/Abstract])) OR (MC1288[Title/Abstract])) OR (Osteotriol[Title/Abstract])) OR (Renatriol[Title/Abstract])) OR (Silkis[Title/Abstract])) OR (Sitriol[Title/Abstract])) OR (Soltriol[Title/Abstract])) OR (Tirocal[Title/Abstract])) OR (20-epi-1alpha,25-dihydroxycholecaliferol[Title/Abstract])) OR (1,25-dihydroxy-20-epi-Vitamin D3[Title/Abstract])) OR (D3, 1,25-dihydroxy-20-epi-Vitamin[Title/Abstract])) OR (1,25(OH)2-20epi-D3[Title/Abstract])) OR (1 alpha, 25-dihydroxy-20-epi-Vitamin D3[Title/Abstract])
#7	#4 OR #5 OR #6
#8	#3 AND #7
